# Molecular profiling of peripheral blood is associated with circulating tumor cells content and poor survival in metastatic castration-resistant prostate cancer

**DOI:** 10.18632/oncotarget.3550

**Published:** 2015-03-12

**Authors:** Mercedes Marín-Aguilera, Òscar Reig, Juan José Lozano, Natalia Jiménez, Susana García-Recio, Nadina Erill, Lydia Gaba, Andrea Tagliapietra, Vanesa Ortega, Gemma Carrera, Anna Colomer, Pedro Gascón, Begoña Mellado

**Affiliations:** ^1^ Translational Genomics Group and Targeted Therapeutics in Solid Tumors Group, Institut d'Investigacions Biomèdiques August Pi i Sunyer (IDIBAPS), Barcelona, Spain; ^2^ Medical Oncology Department, Hospital Clínic, Barcelona, Spain; ^3^ Bioinformatics Platform Department, Centro de Investigación Biomédica en Red en el Área temática de Enfermedades Hepáticas y Digestivas (CIBEREHD), Hospital Clínic, Barcelona, Spain; ^4^ Laboratory of Translational Oncology, Fundació Clínic per a la Recerca Biomèdica, Barcelona, Spain; ^5^ Althia, Barcelona, Spain; ^6^ Medical Oncology Department, Hospital Plató, Barcelona, Spain

**Keywords:** circulating tumor cells, peripheral blood, microarrays, cell search system

## Abstract

The enumeration of circulating tumor cells (CTCs) in peripheral blood correlates with clinical outcome in castration-resistant prostate cancer (CRPC). We analyzed the molecular profiling of peripheral blood from 43 metastatic CRPC patients with known CTC content in order to identify genes that may be related to prostate cancer progression. Global gene expression analysis identified the differential expression of 282 genes between samples with ≥5 CTCs *vs* <5 CTCs, 58.6% of which were previously described as over-expressed in prostate cancer (18.9% in primary tumors and 56.1% in metastasis). Those genes were involved in survival functions such as metabolism, signal transduction, gene expression, cell growth, death, and movement. The expression of selected genes was evaluated by quantitative RT-PCR. This analysis revealed a two-gene model (*SELENBP1* and *MMP9*) with a high significant prognostic ability (HR 6; 95% CI 2.61 - 13.79; P<0.0001). The combination of the two-gene signature plus the CTCs count showed a higher prognostic ability than CTCs enumeration or gene expression alone (P<0.05). This study shows a gene expression profile in PBMNC associated with CTCs count and clinical outcome in metastatic CRPC, describing genes and pathways potentially associated with CRPC progression.

## INTRODUCTION

Prostate cancer (PC) is the second leading cause of death from cancer in men [1]. During the progression of the disease, subgroups of cancer cells reach the circulation and are able to proliferate in distant tissues [2].

Interest has been growing in recent years about circulating tumor cells (CTCs) as leaders in the metastatic process. Because of its great accessibility, the expression profile of peripheral blood is of interest for determining molecular alterations that may justify a more aggressive clinical behavior in patients with higher CTCs count. Studies mostly have focused on the capability of CTCs as biomarkers but also their potential utility in diagnosis, prognosis, and as a marker of treatment effectiveness [3]. These studies are supported by technologies that allow the reproducible detection and quantification of CTCs. These cells can be separated from other hematopoietic cells by physical characteristics such as size and shape, or by biological characteristics such as expression of epithelial or cancer-specific markers. The CellSearch System (Veridex, LLC) is the most widely used CTC-isolation technology in clinical testing. This semi-automated method received U.S. Food and Drug Administration approval for the enumeration of CTCs in whole blood [4, 5]. In castration-resistant PC (CRPC), the quantification of CTCs by CellSearch system has been proved to be clinically relevant. Studies have used this technology to define groups with unfavorable and favorable prognosis among patients with metastatic prostate cancer [6] and for molecular studies of CTCs [6-10]. Furthermore, the decline in the number of CTCs under treatment is a stronger prognostic factor for post-treatment survival than a 50% decline in prostate-specific antigen (PSA) [11]. However, the detection of CTCs by the expression of epithelial specific markers, may have as a consequence that CTCs with different molecular characteristics cannot be detected.

In addition to CTCs, non-tumoral epithelial and circulating hematopoietic cells may also be involved in tumor progression [12]. Indeed, two recently published studies described transcriptional profiles in peripheral blood associated with prognosis in patients with CRPC [13, 14].

The detection of tumor or epithelial markers by reverse-transcriptase polymerase chain reaction (RT-PCR) in the mononuclear cell fraction of peripheral blood (PBMNC) have been widely use as a strategy to detect CTCs in patients with cancer [15]. In the present work we describe a transcriptional profile associated with CTCs count in the PBMNC of patients with metastatic CRPC and molecular pathways that may be associated with CRPC progression. Notably, most of the detected genes in patients with ≥5 CTCs were previously described as over expressed in PC, and specifically 56.1% were over-expressed in PC metastasis. RT-PCR expression of two genes, *SELENBP1* and *MMP9*, together with the CTC count showed a significant prognostic ability in CRPC patients. Overall, our findings support that expression studies in PBMNC in metastatic CRPC patients may translate the biology of the CTCs and may be used to identify patients with a more aggressive clinical behavior.

## RESULTS

### Patients and CTCs count

Seventy-four patients were included in the study. Four of them were excluded from the analysis: one patient that did not accomplish the inclusion criteria and three samples that failed the quality control were discarded for being analyzed. The clinical characteristics of the 70 final patients are shown in Table 1. Patients were prospectively followed from the time of inclusion in the study.

PBMNC from 43 patients were tested for CTC count and microarrays analysis. All the 70 samples were studied by qRT-PCR. Clinical characteristics between patients with ≥5 CTCs and <5CTCs were well balanced (Table 1).

**Figure 1:**
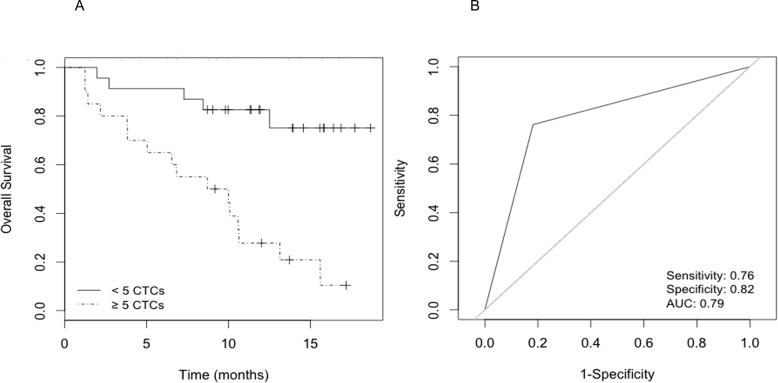
Survival analysis according to CTC count A) Kaplan-Meier curves that estimate the probabilities of overall survival (OS) of CRPC patients with <5 and ≥5 CTCs. The log-rank test was used to assess the statistical difference between the two groups (P<0.001); B) Receiver operating characteristic (ROC) curve for prediction accuracy of ≥5 CTCs content in OS of CRPC.

The median number of CTCs per patient sample was 59 (range 1-899). Specifically, 23 patients (53.4%) presented <5 CTCs (average 1 cell) and 20 patients (46.5%) had ≥5 CTCs (average 126 cells). During a median follow-up period of 12 months, 32 of the 43 patients (74.4%) died. Univariate Cox proportional hazards regression analysis assessed the number of CTCs as a risk factor for overall survival (OS). Patients with <5 CTCs had longer survival relative to patients with ≥5 CTCs by log-rank analysis (Cox HR 5.5; 95%CI, 2-15.3; log-rank P<0.001) (Fig. 1A). The predictive accuracy of the number of CTCs (≥5 CTCs *vs* <5 CTCs) for OS was assessed by the ROC curve (area under curve=0.790) (Fig. 1B).

**Table 1:** Clinical characteristics of patients and CTC count; ^1^CTCs: circulating tumor cells; ^2^PSA: prostatic specific antigen; ^3^AP: Alkaline phosphatase; ^4^LDH: Lactate dehydrogenase; ^5^“events” refer to number of deaths

Patients' characteristics				
CTC^1^ load	<5 CTCs	≥5 CTCs	CTCs non-eval	Total
Number of patients	23	20	27	70
Age (years)				
Median (range)	72 (40.1-78.3)	73.5 (49-83)	67.5 (51-79.8)	66.5 (40.1-83.1)
Gleason N (%)				
≤ 7	9 (39.1)	7 (35)	13 (48.3)	29 (41.5)
8-10	12 (52.2)	10 (50)	12 (44.4)	34 (48.6)
Unknown	2 (9)	3 (15)	2 (7.4)	7 (10)
PSA^2^ (ng/mL)				
Median (range)	24.3 (1.3-1,375)	40.4 (1.8-1,002)	47.5 (1.8-445.6)	57.9 (1.3-1,375)
AP^3^ (U/L)				
Median (range)	180.5 (71-679)	401 (121-5797)	265 (91-1143)	253 (71-5797)
LDH^4^ (U/L)				
Median (range)	408 (253-628)	588 (336-1569)	351 (232-604)	408 (232-1569)
Hemoglobin (g/dL)				
Median (range)	127 (86-153)	112.5 (74-144)	129 (96-156)	127 (74-156)
Previous chemotherapy (%)				
Yes	9 (39.1)	7 (35)	6 (22.2)	22 (31.4)
No	14 (60.9)	13 (65)	21 (77.8)	48 (68.6)
Metastasis				
Bone	18 (46.1)	20 (64.5)	22 (51.2)	60 (53.1)
Visceral	5 (12.8)	1 (3.2)	7 (16.3)	13 (11.5)
Lymph nodes	15 (38.5)	10 (32.3)	12 (27.9)	37 (32.7)
Local relapse	1 (2.6)	0	2 (4.7)	3 (2.7)
CTC number (%)				
0-2	20 (46.5)	-	-	-
3-4	3 (6.9)	-	-	-
5-20	-	6 (13.9)	-	-
21-50	-	9 (20.9)	-	-
>51	-	5 (11.6)	-	-
Follow-up (months)				
Median (range)	12 (1.9-36.8)	12.23 (1.2-35.3)	12 (3.3-26.2)	12 (1.2-36.8)
Number of events^5^ (%)	14 (60.9)	18 (90)	4 (14.8)	36 (51.4)

### Gene expression profiling in peripheral blood mononuclear cells is associated with CTC count

**Figure 2:**
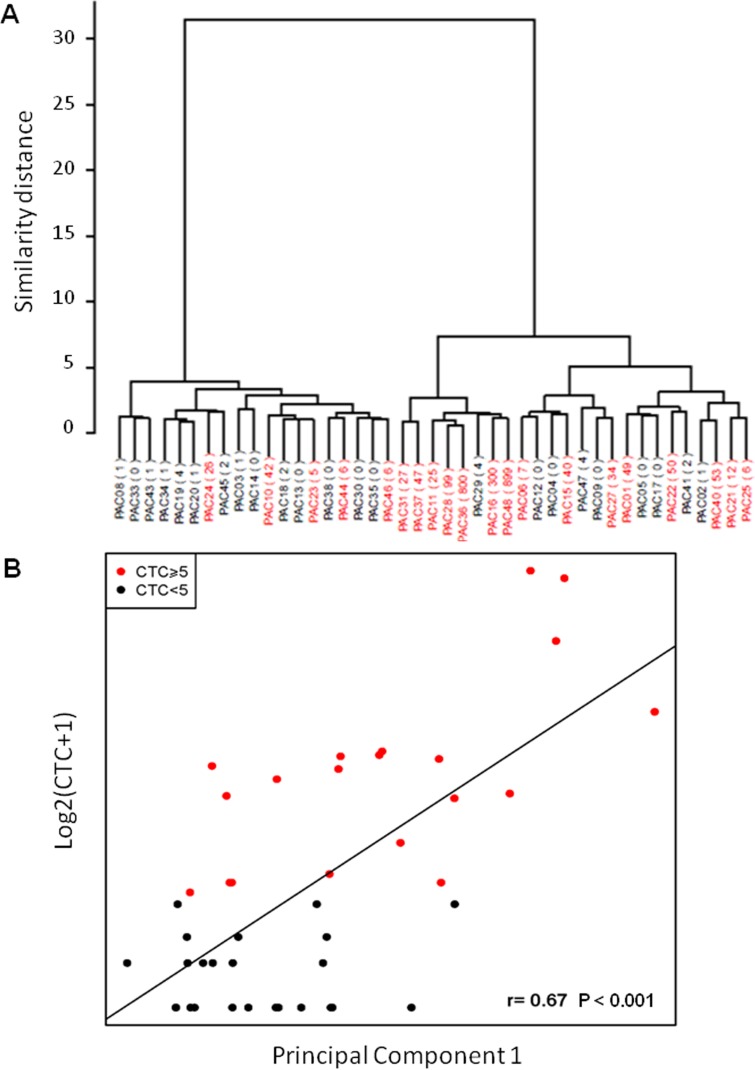
Correlation between gene expression data in PBMNC and CTC count A) Unsupervised clustering grouping patients according to gene expression data; B) Principal component analysis. The Y-axis represents the log2 of the CTC number plus one, and the X-axis is PC1 (Spearman correlation P<0.001).

Hierarchical clustering and principal component analysis algorithms were used to examine the sample groupings in the expression data. Expression profiles grouped samples in ≥5 CTCs and <5 CTCs (Fig. 2A). We found good correlation in the comparison of microarrays results and CTC count (r=0.67, P<0.001, Fig. 2B).

**Figure 3:**
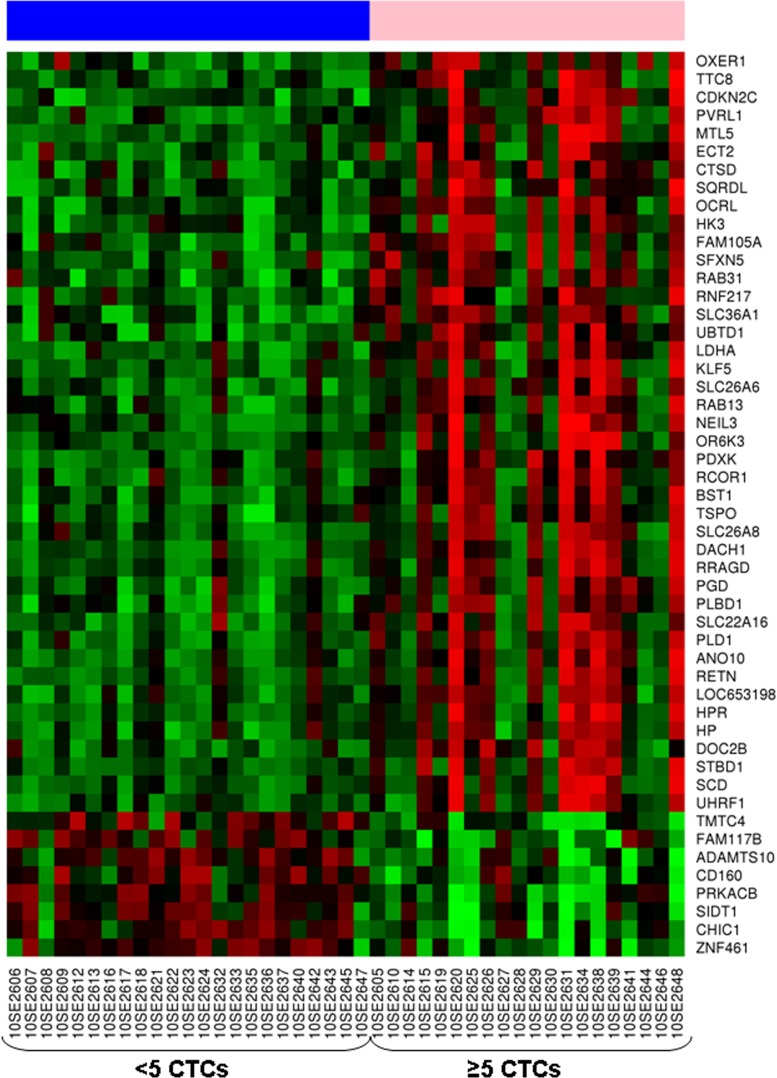
Heatmap representing expression profile of the 50 most differentially expressed genes in samples from castration-resistant prostate cancer patients with ≥5 CTCs compared to those with <5 CTCs (FDR<0.001) Rows represent genes and columns represent hybridized samples. Red pixels: upregulated genes; Green pixels: downregulated genes. The intensity of each color denotes the standardized ratio between each value and the average expression of each gene across all samples.

Microarrays analysis revealed 282 differentially expressed genes (fold change [FC] >│1.5│) between samples with ≥5 CTCs *vs* <5 CTCs. Most of these genes were involved in cellular metabolism, gene expression regulation, and cell morphogenesis, signaling and transport, and were significantly up-regulated in samples with ≥5 CTCs. Genes down-regulated in this group of samples were mostly involved in signal transduction and metabolic process (Supplementary Table S1). The 50 most differentially expressed genes between both groups (FDR<0.001) are showed at Fig. 3. Among the overexpressed genes in samples with ≥5CTCs, 58.6% have previously described as been over-expressed in prostate cancer, specifically 18.9% in primary tumor and 56.1% in metastasis in comparison with normal prostate tissue [16]. The complete list of these genes is shown in Table 2. Reactome pathways analysis revealed significantly deregulated pathways between samples with ≥ or <5 CTCs (P<0.05) (Table 3).

**Table 2:** Genes differentially expressed between ≥5 CTCs and <5 CTCs and also between primary tumor/metastasis and normal prostate tissue according to previous data in the literature [16];*qRT-PCR validated genes

Over-expressed in metastasis vs primary tumor			Over-expressed in primary tumor vs normal prostate tissue	Over-expressed in metastasis vs normal prostate tissue		
Gene symbol			Gene symbol	Gene symbol		
ABCA13*	*HBD*	*SIGLEC14*	*AGAP1*	ABCA13*	*ESCO2*	*OLR1*
*ABCC13*	*HEPACAM2*	*SLC22A16*	*ANKRD18A*	*ABCC13*	*FAM151B*	*OR2W3*
*AFF2*	*HIST1H1B*	*SLC25A39*	*BUB1B*	*AGAP1*	*FAM20A*	*OR6N1*
*AHSP*	*HIST1H3B*	*SLC26A8*	*C15orf42*	*AHSP*	*FAR2*	*OSBP2*
*ALAS2*	*HIST1H3G*	*SLC4A1*	*C17orf66*	*ALAS2*	*FCAR*	*PADI4*
*ANK1*	*HLX*	*SLC6A8*	*CASC5*	*ANK1*	*FCRL6*	*PGLYRP1*
*ANLN*	*HMBS*	*SOX6*	*CCDC125*	*ANLN*	*FOLR3*	*PHOSPHO1*
*ASPM*	*IFIT1B*	*SPTA1*	*CCNB2*	*ARG1*	*FOXM1*	*PLK1*
*AZU1*	*INHBA*	*SPTB*	*CCNE2*	*ASPM*	*GLRX5*	*PPEF1*
*BCL2L15*	*KEL*	*SYTL2*	*CCRN4L*	*AZU1*	*GNLY*	*PRC1*
*BMX*	*KIAA0101*	*TCL1A*	*CD24*	*BCL2L15*	*GPR160*	*PRR13*
*BPI*	*KIF11*	*TCN1*	*CEACAM3*	*BMX*	*GPR84*	*PRTN3*
*BUB1*	*KIF14*	*TNFAIP6*	*CENPF*	*BPI*	*GPR97*	*PTGDR*
*BUB1B*	*KIF15*	*TOP2A*	*CHI3L1*	*BUB1*	*GYPA*	*PTH2R*
*C15orf42*	*KIF20A*	*TPX2*	*CHIT1*	*BUB1B*	*GZMH*	*PVALB*
*C17orf66*	*KIF23*	*TRBV28*	*CKAP2L*	*C15orf42*	*HBD*	*RAP1GAP*
*C19orf59*	*KLRD1*	*TYMS*	*CLEC5A*	*C15orf54*	*HBM*	*RETN*
*C19orf77*	*LOC285696*	*UHRF1*	*CRISP2*	*C17orf66*	*HEPACAM2*	*RHAG*
*CAMK2N1*	*LRRC4*	*YOD1*	CRISP3*	*C19orf59*	*HIST1H1B*	*RRM2*
*CAMP*	*LRRN1*	*ZNF788*	*DLGAP5*	*C19orf77*	*HIST1H3B*	*RUNDC3A*
*CASC5*	*MGAM*		ERG*	*CA1*	*HIST1H3G*	*S100P*
*CCNA2*	*MKI67*		*FAM151B*	*CAMP*	*HLA-DRB5*	*SERPINB10*
*CCNB2*	*MLNR*		*FOXM1*	*CASC5*	*HLX*	*SERPINB2*
*CCNE2*	MMP8*		*FZD5*	*CCNA2*	*HMBS*	*SIGLEC14*
*CD160*	MMP9*		*GCA*	*CCNB2*	HP*	*SIGLEC5*
*CDK1*	*MPO*		*GPR160*	*CCNE2*	HPR*	*SLC22A16*
*CEACAM3*	*MS4A3*		*GPR84*	*CCRN4L*	*HTRA3*	*SLC25A39*
*CEACAM4*	*MYL4*		*GZMH*	*CD160*	*IFIT1B*	*SLC26A8*
*CEACAM8*	*MYO6*		*HIST1H3B*	*CD3G*	*INHBA*	*SLC27A2*
*CENPF*	*NLRC4*		HP*	*CDK1*	*KEL*	*SLC28A3*
*CIT*	*NUSAP1*		HPR*	*CEACAM3*	*KIAA0101*	*SLC4A1*
*CKAP2L*	*OR2W3*		*ICA1*	*CEACAM4*	*KIF11*	*SLC6A8*
*CRISP2*	*OR6N1*		*KIF11*	*CEACAM8*	*KIF14*	*SLC6A9*
*CTSG*	*OSBP2*		*KIF20A*	*CENPF*	*KIF15*	*SOX6*
*CYP4F3*	*PADI4*		*MBOAT2*	*CHI3L1*	*KIF20A*	*SPTA1*
*DEFA4*	*PGLYRP1*		*MKI67*	*CHIT1*	*KIF23*	*SPTB*
*DHRS9*	*PHOSPHO1*		MMP9*	*CIT*	*LIN7A*	*SUCNR1*
*DLGAP5*	*PIWIL4*		*MYB*	*CKAP2L*	*LOC100289137*	*SYTL2*
*DTL*	*PLK1*		*MYO6*	*CLEC5A*	*LRRC4*	*TCL1A*
*E2F2*	*PPEF1*		*NUSAP1*	*CRISP2*	*LRRN1*	*TCN1*
*E2F8*	*PRC1*		*OLR1*	CRISP3*	*MBOAT2*	*TNFAIP6*
*ECRP*	*PRTN3*		*PPEF1*	*CTSG*	*MGAM*	*TNFRSF10C*
*EPB42*	*PTCH1*		*PRC1*	*CYP4F3*	*MKI67*	*TOP2A*
*ESCO2*	*PTGDR*		*PRR13*	*DEFA4*	*MLNR*	*TPX2*
*FAM151B*	*PVALB*		*PTH2R*	*DHRS9*	MMP8*	*TRBV28*
*FAR2*	*RETN*		*RAP1GAP*	*DLGAP5*	MMP9*	*TRIM58*
*FCAR*	*RHAG*		*RRM2*	*DTL*	*MPO*	*TYMS*
*FOXM1*	*RNASE2*		*SIGLEC14*	*E2F2*	*MS4A3*	*UHRF1*
*GNLY*	*RRM2*		*SIGLEC5*	*E2F8*	*MYL4*	*VSTM1*
*GPR84*	*RUNDC3A*		*SLC27A2*	*ECRP*	*MYO6*	*YOD1*
*GPR97*	*SAMD3*		*SLC28A3*	*ELANE*	*NCALD*	*ZNF788*
*GYPA*	*SERPINB10*		*TOP2A*	*EPB42*	*NLRC4*	
*GZMH*	*SERPINB2*	**	*TPX2*	ERG*	*NUSAP1*	

**Table 3:** Reactome pathways analysis showing significantly deregulated pathways between samples with ≥5 CTCs vs <5 CTCs (P<0.05)

Genes involved	N genes at pathway	REACT_ID	Name of the event
*RFC2, HSPA2, HIST2H2AA4*	103	REACT_22172	Chromosome Maintenance
*CYP24A1, PTGIS, MGST2*	138	REACT_13433	Biological oxidations
*CYP24A1, PTGIS*	49	REACT_13567	Cytochrome P450 - arranged by substrate type
*HIST2H2AA4*	56	REACT_7970	Telomere Maintenance
*HSPA2, RFC2, HIST2H2AA4*	57	REACT_75792	Meiotic Synapsis

### Gene expression profiling in peripheral blood mononuclear cells is associated with overall survival

Differential expression analysis revealed a large set of genes (False Discovery Rate (FDR)<5; signal expression median >7) that were significantly up- (840 genes) and downregulated (948 genes) and significantly correlated with lower survival. The most frequent biological functions in which overexpressed genes were involved were cell movement, transport, metabolism and signaling. Pathways analysis revealed a set of up- (162) and downregulated (239) pathways related to OS (Supplementary Table S2). The identified pathways can be grouped into a limited number of categories: metabolism, apoptosis, DNA damage and repair, protein degradation, immune system, signal transduction, cell transport, cell growth, gene expression, protein organization and homeostasis. Among those associated with lower survival, 100 genes were also deregulated in samples with ≥5 CTCs (78 overexpressed and 22 downexpressed) (Supplementary Table S3).

### Expression data from selected genes were validated by qRT-PCR

To determine the robustness of cDNA microarrays, we performed qRT-PCR of a selected group of ten genes among the top 40 more differentially expressed between samples with ≥5 and <5 CTCs count and/or related to lower OS. These genes were *HP*, *CRISP3*, *MMP9*, *ABCA13*, *MMP8*, *OLFM4*, *SELENBP1*, *CEACAM1*, *HPR* and *ERG.* Differential gene expression determined by microarrays was confirmed in all genes except for *HP* (90%).

### A two-gene signature predicts overall survival in metastatic CRPC

QRT-PCR data from the 9 out of 10 technically top differentially expressed validated genes was used to test, individually and combined, selected genes as predictors of OS. This initial analysis revealed that the expression of *CRISP3*, *MMP9*, *ABCA13*, *MMP8*, *OLFM4*, *SELENBP1* and *CEACAM1* significantly correlated with lower OS (P<0.05) (Table 4A). These significant genes were tested in a set of PBMNC RNA samples from additional 27 patients. A global OS analysis was performed in the whole series (N=70) confirming the predictive value of these genes in the univariate analysis (Table 4B).

**Table 4:** Univariate analysis of RT-PCR validated genes for predicting OS in: A) the cohort of patients with known CTCs count (N:43); B) the whole series of patients (N:70)

A	OS-initial series (N:43)			
	Hazard ratio (95% CI)	P-value	Odds ratio (95% CI)	P-value
*ABCA13*	1.40 (1.15 - 1.70)	0.000673	1.60 (1.16 - 2.20)	0.004
*CEACAM*	1.47 (1.18 - 1.82)	0.000556	1.73 (1.18 - 2.53)	0.005
*CRISP3*	1.40 (1.16 - 1.68)	0.000339	1.53 (1.15 - 2.03)	0.003
*MMP8*	1.44 (1.19 - 1.74)	0.000187	1.60 (1.18 - 2.17)	0.002
*MMP9*	1.38 (1.15 - 1.65)	0.000593	1.52 (1.15 - 2.00)	0.003
*OLFM4*	1.46 (1.20 - 1.78)	0.000167	1.64 (1.18 - 2.28)	0.004
*SELENBP1*	1.21 (1.06 - 1.39)	0.00501	1.44 (1.09 - 1.90)	0.009

B	OS-global series (N:70)			
	Hazard ratio (95% CI)	P-value	Odds ratio (95% CI)	P-value
*ABCA13*	1.23 (1.08 - 1.41)	0.00224	1.40 (1.13 - 1.81)	0.00399
*CEACAM*	1.26 (1.09 - 1.47)	0.00232	1.49 (1.17 - 1.98)	0.0027
*CRISP3*	1.18 (1.04 - 1.33)	0.00771	1.29 (1.07 - 1.61)	0.0138
*MMP8*	1.22 (1.09 - 1.37)	0.000655	1.41 (1.16 - 1.77)	0.00152
*MMP9*	1.34 (1.15 - 1.57)	0.000151	1.47 (1.19 - 1.86)	0.000644
*OLFM4*	1.26 (1.10 - 1.44)	0.00102	1.38 (1.13 - 1.73)	0.00278
*SELENBP1*	1.23 (1.09 - 1.39)	0.000907	1.36 (1.14 - 1.69)	0.00184

**Figure 4:**
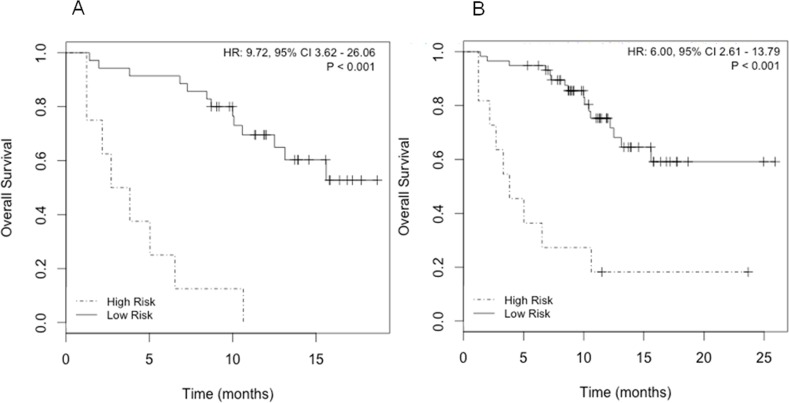
Survival analysis according to *SELENBP1* and *MMP9*expression A) Kaplan-Meier curves in patients with known CTCs count (N=43); B) Kaplan-Meier curves in the whole series of patients (N=70).

**Figure 5:**
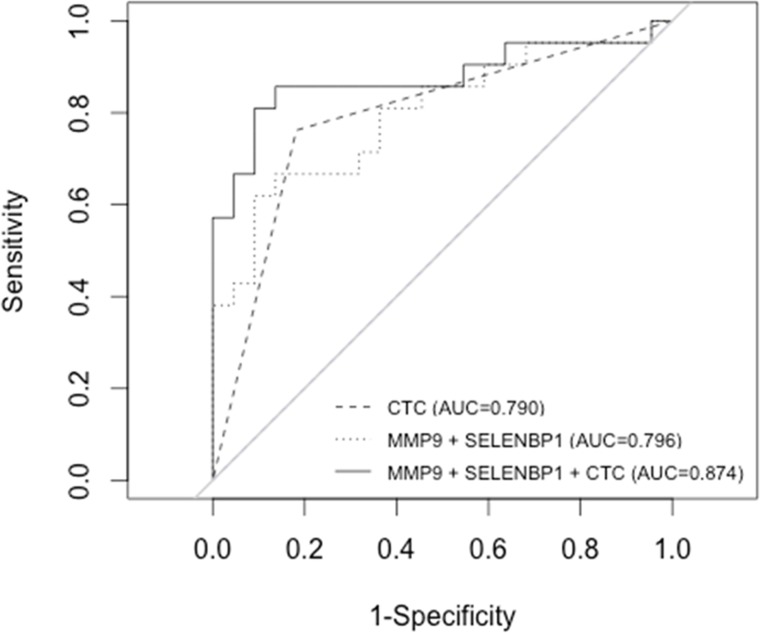
Receiver operating characteristic (ROC) curves illustrating CTCs count and two-gene signature (*MMP9+SELENBP1*) alone and combined for the prediction of OS

The combination of *SELENBP1* and *MMP9* gene expression data was the best gene signature to indicate poorer prognosis, since elevated expression of both markers significantly correlated with a lower OS (Fig. 4). The predictive accuracy of the two-gene signature for OS was assessed by the ROC curve (AUC: 0.79) (Fig. 5). Taking into account the whole series of patients (N=70), the predictive accuracy of the two-gene expression model was similar than in the subset of 43 initial patients (AUC: 0.77; data not shown).

Cox regression models of *SELENBP1* and *MMP9* gene expression, and of the dicotomic CTCs count (≥5 and <5-CTCs) were compared and not statistically significant differences were found between both models (P=0.47). A combined model including both two-gene expression and CTCs count resulted to be a better predictive model for OS (AUC: 0.87) than individually by ROC analysis (Fig. 5). Statistically significant differences between the combined model and the individual parameters were found (*MMP9*+*SELENBP1* vs *MMP9*+*SELENBP1*+CTCs count P<0.05; CTCs count vs *MMP9*+*SELENBP1*+CTCs count P<0.05).

In a multivariable Cox model adjusted for the expression of the two-gene signature (*MMP9*+*SELENBP1)*, CTCs count and clinical characteristics of high risk progression (PSA, alkaline phosphatase, lactate dehydrogenase, hemoglobin levels at time of the sample extraction), the expression of the two-gene signature remained independently predictive of shorter OS (HR 14.53; 95% CI, 1.91 - 110.8; P<0.01); the CTCs count was also predictive of shorter OS (HR 6.94; 95% CI, 1.76 - 27.34; P<0.01). In the global series CTCs count was not included in the multivariate model, because of the lack of data in 27 samples, and the two-gene signature remained independently predictive of shorter OS (HR 3.42; 95% CI, 1.11 - 10.52; P<0.05) (Table 5).

**Table 5:** Multivariable Cox models adjusted for CTCs (circulating tumor cells), *SELENBP1+MMP9*expression, PSA (prostatic specific antigen), AP (Alkaline phosphatase), LDH (Lactate dehydrogenase) and presence of visceral metastases

Patients with CTC count (N=43)				
	HR	95% CI		P-value
*MMP9 - SELENBP1*	14.53	1.91	110.75	0.0098
Number of CTC (≥ or < 5)	6.95	1.76	27.34	0.0056
PSA	0.99	0.99	1	0.7737
FA	1	1	1	0.02
LDH	0.99	0.99	1	0.3725
Hb	0.97	0.94	1	0.1001
Visceral metastases	2.89	0.45	18.63	0.2638

All patients (N=70)				
	HR	95% CI		P-value
*MMP9 - SELENBP1*	3.42	1.11	10.52	0.0321
PSA	1	0.99	1	0.6633
FA	0.99	0.99	1	0.237
LDH	1	0.99	1	0.2737
Hb	0.96	0.93	0.99	0.0146
Visceral metastases	1.08	0.34	3.47	0.8901

## DISCUSSION

The CTCs are a fundamental prerequisite to the spread of solid cancers, however, the metastatic process appears to be inefficient since only a subpopulation of CTCs has the potential to initiate clonal metastatic lesions, which leads ultimately to the death of patients [17]. The study of the biological characteristics of cells responsible for tumor aggression is one of the challenges for CTC research.

The current study identified genes and molecular pathways that represent biological differences between groups of metastatic CRPC patients with different CTC load and prognosis by using comprehensive gene expression analysis of blood samples. We observed differential expression of 282 genes between samples with ≥5 CTCs and <5 CTCs (FC >│1.5│). It is important to point out that most of the differential expressed genes had been previously described as associated with PC [16], supporting that molecular alterations present in tumors may be also detected in peripheral blood. Those genes were involved in survival functions such as metabolism, signal transduction, gene expression, and cell growth, death, and movement. Specifically, among the pathways deregulated we found chromosome and telomere maintenance, which is a hallmark of cancer cells compared to normal cells. Also it is of note the involvement of biological oxidation which constitutes the basis for obtaining energy during tumor growth and metastasis process [18]. The deregulation of these pathways is consistent with a previously reported gene expression meta-analysis that identified biological pathways involved in metastasis of breast cancer [19].

We also describe genes and pathways related to lower OS, which are involved in cell survival functions such as metabolism, signal transduction, transport, and movement, providing biological information that may be relevant in the understanding of the process on CRPC progression. Thus, in the RT-PCR validation study we identified seven differentially expressed genes that correlated with lower OS: *CRISP3*, *MMP9*, *ABCA13*, *MMP8*, *OLFM4*, *SELENBP1* and *CEACAM1*. Of note, high levels of *CRISP3* expression have been previously found in CRPC and metastases [20]. Moreover, metallopeptidases are known, not only to contribute to cancer progression and invasion, but also to signaling pathways that control cell growth, inflammation, or angiogenesis [21]. Specifically, *MMP9* is a known *ERG* target [22], which is one of the genes significantly upregulated in samples with ≥5 CTCs. Further, *ABCA13* gene belongs to the ATP-binding cassette (ABC) family of transmembrane transporters and has been observed to be significantly deregulated in the castration-resistant human cell lines DU-145 that are resistant to docetaxel [23]. *OLFM4* promotes S phase transition in cancer cells as well as being associated with cell adhesion and metastasis [24]. *SELENBP1* gene is expressed in LNCaP cells and is reversibly downregulated by androgen. However, it is not expressed by either of two androgen-insensitive human lines, PC-3 and DU-145 [25]. The exact function of *SELENBP1* remains unknown. Finally, *CEACAM1* epithelial marker has been related with increased vascularization of prostate cancer [26].

The validation of this seven-gene model in a wider cohort demonstrated the strong prognostic significance of the combination of *SELENBP1* and *MMP9* gene expression. Although the described gene signature did not increase the prognostic significance of CTCs enumeration alone, it adds prognostic ability when it was used in combination with CTCs count. Interestingly, *MMP9* and *SELENBP1* were also upregulated in PBMCs from the short-term survivors patients included in a microarrays study from Komatsu et al. [27]. Authors suggested the relevance of the immune system in CTCs load and, consequently, in clinical and biological behavior of prostate cancer.

Two studies described whole-blood RNA transcript-based prognostic models in metastatic CRPC. Ross et al. described a six-gene signature (consisting of *ABL2*, *SEMA4D*, *ITGAL*, and *C1QA*, *TIMP1*, *CDKN1A*) that separated patients with CRPC into two risk groups: a low-risk group with a not reached median survival (more than 34.9 months) and a high-risk group with a median survival of 7.8 months (P<0.0001), that was validated in an independent series of 140 patients. The authors concluded that the six-gene model suggests a possible deregulation of the immune system, a finding that warrants further study [13]. On the other hand, Olmos et al. identified a nine-gene signature associated with worse OS. Expression profiles were analyzed with Bayesian latent process decomposition (LPD) and patients were stratified into distinct prognostic groups, with different survival: the LPD1, with a median OS of 9.2 months *vs* 21.6 months in the non-LPD1 [7.5-35.6]; P=0.001).

In conclusion, we describe a transcriptional profile in PBMNC associated with CTCs count that shows deregulated pathways that may contribute to PC progression. The knowledge of the molecular alterations associated with CTC load in peripheral blood has revealed genes, which are likely to be responsible for tumor aggressiveness and are potential targets for the development of future treatments.

## METHODS

### Patients and samples

Inclusion criteria was histologically or citologically documented prostate cancer diagnosis, stage IV, that where considered to be in a CRPC status defined as the Prostate Cancer Clinical Trials Working Group (PCWG2) criteria [28]. Patients were included at the time of progression or before starting a new antitumor therapy. Disease progression was defined as rising levels of PSA or radiographic criteria, following the PCWG2 criteria [28].

The study was designed as a prospective study. Patients were prospectively followed from the time of study inclusion until death or last visit. OS was determined from the date of CTC determination to the date of death or last follow-up visit. The institutional committee on human experimentation approved this study and written informed consent was obtained from all patients.

The design of this study comprised a first stage, were an initial cohort of patients were tested for CTCs count and microarrays analysis, and a second stage, were a selected set of genes were validated by RT-qPCR in the same cohort plus in a new series of patients. Gene expression data were correlated with clinical outcome.

### CTCs enumeration

CTCs were counted and isolated as described previously [6, 29]. Briefly, 7.5 mL of peripheral blood was collected in CellSave Preservative Tubes (Veridex, LLC) following manufacturer's instructions. The first 5 mL of blood were discarded to avoid epithelial contamination by the skin during venipuncture. Samples were kept at room temperature for up to 96 hours until processing. CTCs were enriched by immunomagnetic isolation based on the expression of epithelial cellular adhesion molecules (EpCAM) using the CellSearhc Circulating Tumor Cell Kit with an automated cell processor (CellTracks Analyzer II; Veridex, LLC). The ferromagnetic reagent was conjugated with a phycoerythrin fluorochrome. Labeled cells were resuspended in a CellTracks Magnetic cartouche and analyzed by a semi-automated fluorescence cell reader (CellTracks Analyzer II; Veridex, LLC). CD45 was used to determine potential contamination of blood cells with leucocytes. The CTCs were identified by their DAPI (nuclear) and EpCAM expression in the absence of CD45-staining.

### Total RNA extraction

Five mL of peripheral blood samples were collected into Monovette EDTA–containing Vacutainers (Sarstedt). Blood specimens were layered onto 4 mL of Ficoll-Paque (GE Healthcare Life Sciences). Mononuclear cells were isolated and total RNA was extracted using Trizol Reagent (Invitrogen Life Technologies) according to manufacturer's instructions. RNA was quantified by ND-1000 Spectrophotometer (Nanodrop Technologies).

### cDNA Microarray

For microarrays hybridization, RNAs were purified using the RNeasy Micro kit (Qiagen), and quality were measured by Bioanalyzer technology. cDNA was generated from 300 ng of total RNA using the Ambion WT Expression Kit (Applied Biosystems) and according to manufacturer's instructions.

Fragmented, labeled and amplified cDNA was hybridized to the Human Gene 1.1 ST Array (Affymetrix), which represents approximately 33,000 probe sets. Wash, rinse and scanning of the arrays was performed according to manufacturer's instructions. Quality of the microarray was assessed by dChip [30] and Expression Console (Affymetrix) softwares.

Raw expression data from microarrays were normalized using the robust multiarray algorithm [31] with a custom probe set definition that mapped probes to Entrez Gene IDs (HuGene11stv1_Hs_ENTREZG) [32]. To identify differentially expressed genes between the different microarray study groups we employed Significant Analysis of Microarray [33]. One thousand permutations of the data were used to estimate the False Discovery Rate (FDR) and to select differentially expressed genes.

The CEL files and RMA values were deposited on Gene Expression Omnibus (GSE66532).

### qRT-PCR

One μg of total RNA was reverse transcribed using the High Capacity cDNA Archive Kit (Life Technologies) following manufacturer's instructions. A real-time quantitative reverse transcription PCR (qRT-PCR) was performed in a StepOnePlus Real-Time PCR system (Life Technologies) according to the manufacturer's recommendations. Data was acquired using SDS Software 1.4. Amplification reactions were performed by duplicate. Expression values were based on the quantification cycle (Cq) from target genes relative to the Cq of *ACTB* endogenous gene. Relative expression with respect to each reference group studied was reported as LogRatio. Commercial codes for primers and probes were used to amplify target genes (Life Technologies).

### Differential gene expression analysis and validation

The number of CTCs was correlated with transcriptomic profile of genes with a median log2 intensity of 6 (9509 genes, 48.5% of total genes). Patients were clustered using spearman correlation and complete distance method. Principal Component Analysis (PCA) was used to test variability of gene expression according to CTC number. Pathways analysis was performed using Reactome, an open source, peer-reviewed database of human pathways and processes [34].

Ten among the forty most differentially expressed genes between the <5 and ≥5 CTCs groups were selected for further validation in PBMNC samples from the same patients studied by microarrays. Among them, those who significantly predict OS were analyzed in a new series of samples, using qRT-PCR.

### Statistical analysis methods

Continuous CTC counts data were converted to a binary classification (≥5 CTCs *vs* <5 CTCs). Kaplan-Meier survival curves were generated and evaluated by log-rank test. For multivariate logistic regression, the Akaike information criterion (AIC)-based backward selection was used to drop insignificant terms [35].

The CTC count data were further analyzed with a receiver operating characteristic (ROC) curve to estimate a cut-off value that maximized sensitivity and specificity. The relation between gene expression and clinical outcome was analyzed with Cox proportional hazard model. The stepwise Cox regression model for multivariate analysis was used to evaluate such association. P-value ≤0.05 was considered. The best combination of two genes was selected. The resulting two-gene model was tested on the initial set of 43 patients (training set) and with additional 27 patients. This analysis was done on a masked basis. Individual model risk scores were computed for each individual patient according to the prespecified coefficients established in the training set for the two-gene model. We developed a cutpoint value (Youden Index derived from Risk scores and status) in the training set that was applied to split the samples into two groups according to prognostic risk. Kaplan-Meier survival curves were generated for the two groups on the basis of the prespecified cutpoint. We use the dichotomous variable representing the two risk groups being used as a single covariate.

All computations were performed using R statistical software [36].

## SUPPLEMENTARY MATERIALS, TABLES


