# Correction: Molecular profiling of peripheral blood is associated with circulating tumor cells content and poor survival in metastatic castration-resistant prostate cancer

**DOI:** 10.18632/oncotarget.17148

**Published:** 2017-04-17

**Authors:** Mercedes Marín-Aguilera, Òscar Reig, Juan José Lozano, Natalia Jiménez, Susana García-Recio, Nadina Erill, Lydia Gaba, Andrea Tagliapietra, Vanesa Ortega, Gemma Carrera, Anna Colomer, Pedro Gascón, Begoña Mellado

**Present**: The acknowledgement information is incomplete.

Correct: Additional acknowledgements are shown below.

**ACKNOWLEDGEMENTS**

This work was co-financed by European Development Regional Fund (ERDF).

Original article: Oncotarget. 2015; 6:10604-16. doi: 10.18632/oncotarget.3550

