# Supplementary material for: Molecular profiling of peripheral blood is associated with circulating tumor cells content and poor survival in metastatic castration-resistant prostate cancer
Source: Oncotarget. 2015 Mar 12;6(12):10604–16. doi: 10.18632/oncotarget.3550 (PMC4496379; doi:10.18632/oncotarget.3550)
Supplement: Supplementary file 1 [file oncotarget-06-10604-s001.pdf]

Molecular profiling of peripheral blood is associated with circulating tumor cells content and poor survival in metastatic castration-resistant prostate cancer

Supplementary Material

Supplementary Table S1: Genes differentially expressed between ≥5 CTCs and <5 CTCs and their corresponding Fold change; FC: Fold change; FDR: False discovery rate.

Probe ID	Gene symbol	Gene name	FC	FDR (%)
3240_at	*HP*	haptoglobin	4.093	0.3916
1116_at	*CHI3L1*	chitinase 3-like 1 (cartilage glycoprotein-39)	3.580	0.3916
4973_at	*OLR1*	oxidized low density lipoprotein (lectin-like) receptor 1	3.482	0.3916
10321_at	*CRISP3*	cysteine-rich secretory protein 3	3.418	1.0624
4318_at	*MMP9*	matrix metallopeptidase 9 (gelatinase B, 92kDa gelatinase, 92kDa type IV collagenase)	3.288	0.7929
6521_at	*SLC4A1*	solute carrier family 4, anion exchanger, member 1 (erythrocyte membrane protein band 3, Diego blood group)	3.283	2.5934
57126_at	*CD177*	CD177 molecule	3.271	0.6156
2993_at	*GYPA*	glycophorin A (MNS blood group)	3.243	2.5934
2994_at	*GYPB*	glycophorin B (MNS blood group)	3.192	2.3410
154664_at	*ABCA13*	ATP-binding cassette, sub-family A (ABC1), member 13	3.124	1.0624
383_at	*ARG1*	arginase, liver	3.116	1.0624
51327_at	*AHSP*	alpha hemoglobin stabilizing protein	3.110	2.5934
820_at	*CAMP*	cathelicidin antimicrobial peptide	3.090	0.3916
439996_at	*IFIT1L*	interferon-induced protein with tetratricopeptide repeats 1-like	3.066	2.3410
4317_at	*MMP8*	matrix metallopeptidase 8 (neutrophil collagenase)	3.007	1.7031
6590_at	*SLPI*	secretory leukocyte peptidase inhibitor	2.917	0.6156
4051_at	*CYP4F3*	cytochrome P450, family 4, subfamily F, polypeptide 3	2.890	0.6156
10562_at	*OLFM4*	olfactomedin 4	2.852	2.2070

759_at	*CA1*	carbonic anhydrase I	2.843	4.4952
1118_at	*CHIT1*	chitinase 1 (chitotriosidase)	2.839	1.0142
212_at	*ALAS2*	aminolevulinate, delta-, synthase 2	2.836	4.0068
306_at	*ANXA3*	annexin A3	2.818	1.0624
8991_at	*SELENBP1*	selenium binding protein 1	2.807	2.5934
4057_at	*LTF*	lactotransferrin	2.782	1.9145
6947_at	*TCN1*	transcobalamin I (vitamin B12 binding protein, R binder family)	2.761	1.1053
5273_at	*SERPINB10*	serpin peptidase inhibitor, clade B (ovalbumin), member 10	2.732	0.6156
5004_at	*ORM1*	orosomucoid 1	2.716	0.7929
634_at	*CEACAM1*	carcinoembryonic antigen-related cell adhesion molecule 1 (biliary glycoprotein)	2.694	1.0142
2038_at	*EPB42*	erythrocyte membrane protein band 4.2	2.645	2.5934
7111_at	*TMOD1*	tropomodulin 1	2.620	1.1053
57633_at	*LRRN1*	leucine rich repeat neuronal 1	2.576	0.3916
1991_at	*ELANE*	elastase, neutrophil expressed	2.545	1.1053
8993_at	*PGLYRP1*	peptidoglycan recognition protein 1	2.511	1.1053
1088_at	*CEACAM8*	carcinoembryonic antigen-related cell adhesion molecule 8	2.506	2.3410
3250_at	*HPR*	haptoglobin-related protein	2.501	0.3916
2078_at	*ERG*	v-ets erythroblastosis virus E26 oncogene homolog (avian)	2.493	0.3916
671_at	*BPI*	bactericidal/permeability-increasing protein	2.449	1.7031
222487_at	*GPR97*	G protein-coupled receptor 97	2.437	0.3916
1511_at	*CTSG*	cathepsin G	2.437	1.9145
4680_at	*CEACAM6*	carcinoembryonic antigen-related cell adhesion molecule 6 (non-specific cross reacting antigen)	2.396	2.5934
81442_at	*OR6N2*	olfactory receptor, family 6, subfamily N, member 2	2.391	0.3916
3624_at	*INHBA*	inhibin, beta A	2.374	0.3916

5657_at	*PRTN3*	proteinase 3	2.372	0.6156
566_at	*AZU1*	azurocidin 1	2.371	1.5110
100133941_at	*CD24*	CD24 molecule	2.361	1.0142
1669_at	*DEFA4*	defensin, alpha 4, corticostatin	2.330	4.0068
4353_at	*MPO*	myeloperoxidase	2.319	1.9145
653198_at	*NA*	NA	2.317	0.3916
6286_at	*S100P*	S100 calcium binding protein P	2.299	1.0624
3680_at	*ITGA9*	integrin, alpha 9	2.298	0.3916
3934_at	*LCN2*	lipocalin 2	2.282	1.0142
3045_at	*HBD*	hemoglobin, delta	2.271	2.3410
3042_at	*HBM*	hemoglobin, mu	2.257	4.0068
8972_at	*MGAM*	maltase-glucoamylase (alpha-glucosidase)	2.238	1.1053
391114_at	*OR6K3*	olfactory receptor, family 6, subfamily K, member 3	2.227	0.3916
5909_at	*RAP1GAP*	RAP1 GTPase activating protein	2.199	5.4721
56729_at	*RETN*	resistin	2.171	0.3916
128372_at	*OR6N1*	olfactory receptor, family 6, subfamily N, member 1	2.165	0.3916
6037_at	*RNASE3*	ribonuclease, RNase A family, 3 (eosinophil cationic protein)	2.155	1.5110
116369_at	*SLC26A8*	solute carrier family 26, member 8	2.123	0.3916
932_at	*MS4A3*	membrane-spanning 4-domains, subfamily A, member 3 (hematopoietic cell-specific)	2.119	1.7031
284422_at	*C19orf77*	chromosome 19 open reading frame 77	2.119	1.0142
26499_at	*PLEK2*	pleckstrin 2	2.118	1.5110
2334_at	*AFF2*	AF4/FMR2 family, member 2	2.113	0.6156
148534_at	*TMEM56*	transmembrane protein 56	2.106	1.7031
10900_at	*RUNDC3A*	RUN domain containing 3A	2.095	3.3639

23601_at	*CLEC5A*	C-type lectin domain family 5, member A	2.090	0.6156
7180_at	*CRISP2*	cysteine-rich secretory protein 2	2.068	0.3916
2235_at	*FECH*	ferrochelatase (protoporphyria)	2.051	2.9821
64078_at	*SLC28A3*	solute carrier family 28 (sodium-coupled nucleoside transporter), member 3	2.050	0.3916
162466_at	*PHOSPHO1*	phosphatase, orphan 1	2.050	6.4318
4070_at	*TACSTD2*	tumor-associated calcium signal transducer 2	2.043	1.0624
8218_at	*CLTCL1*	clathrin, heavy chain-like 1	2.028	0.3916
7298_at	*TYMS*	thymidylate synthetase	2.026	0.3916
11001_at	*SLC27A2*	solute carrier family 27 (fatty acid transporter), member 2	2.003	0.3916
54443_at	*ANLN*	anillin, actin binding protein	2.001	0.6156
118932_at	*ANKRD22*	ankyrin repeat domain 22	1.998	1.7031
253012_at	*HEPACAM2*	HEPACAM family member 2	1.997	8.3194
1602_at	*DACH1*	dachshund homolog 1 (Drosophila)	1.997	0.3916
51312_at	*SLC25A37*	solute carrier family 25, member 37	1.994	1.5110
4288_at	*MKI67*	antigen identified by monoclonal antibody Ki-67	1.971	0.6156
688_at	*KLF5*	Kruppel-like factor 5 (intestinal)	1.971	0.3916
660_at	*BMX*	BMX non-receptor tyrosine kinase	1.965	1.0142
643332_at	*LOC643332*	similar to Nonsecretory ribonuclease precursor (Ribonuclease US) (Eosinophil-derived neurotoxin) (RNase UpI-2) (Ribonuclease 2) (RNase 2)	1.955	0.3916
10112_at	*KIF20A*	kinesin family member 20A	1.937	0.3916
9914_at	*ATP2C2*	ATPase, Ca++ transporting, type 2C, member 2	1.936	0.3916
23762_at	*OSBP2*	oxysterol binding protein 2	1.934	3.3639
6708_at	*SPTA1*	spectrin, alpha, erythrocytic 1 (elliptocytosis 2)	1.931	2.5934
25853_at	*DCAF12*	DDB1 and CUL4 associated factor 12	1.930	1.5110

54682_at	*MANSC1*	MANSC domain containing 1	1.919	1.7031
8987_at	*STBD1*	starch binding domain 1	1.914	0.3916
6005_at	*RHAG*	Rh-associated glycoprotein	1.910	3.4989
6535_at	*SLC6A8*	solute carrier family 6 (neurotransmitter transporter, creatine), member 8	1.907	2.9821
7153_at	*TOP2A*	topoisomerase (DNA) II alpha 170kDa	1.906	0.3916
23569_at	*PADI4*	peptidyl arginine deiminase, type IV	1.900	0.6156
55553_at	*SOX6*	SRY (sex determining region Y)-box 6	1.898	4.9964
51629_at	*SLC25A39*	solute carrier family 25, member 39	1.898	2.5934
440603_at	*BCL2L15*	BCL2-like 15	1.897	0.3916
6518_at	*SLC2A5*	solute carrier family 2 (facilitated glucose/fructose transporter), member 5	1.895	0.6156
7357_at	*UGCG*	UDP-glucose ceramide glucosyltransferase	1.890	0.3916
253650_at	*ANKRD18A*	ankyrin repeat domain 18A	1.889	0.6156
9768_at	*KIAA0101*	KIAA0101	1.877	0.3916
84418_at	*C5orf32*	chromosome 5 open reading frame 32	1.876	0.3916
2335_at	*FN1*	fibronectin 1	1.851	2.9821
6319_at	*SCD*	stearoyl-CoA desaturase (delta-9-desaturase)	1.846	0.3916
55137_at	*FIGN*	fidgetin	1.840	2.2070
85413_at	*SLC22A16*	solute carrier family 22 (organic cation/carnitine transporter), member 16	1.840	0.3916
53831_at	*GPR84*	G protein-coupled receptor 84	1.840	0.7929
669_at	*BPGM*	2,3-bisphosphoglycerate mutase	1.832	4.4952
5475_at	*PPEF1*	protein phosphatase, EF-hand calcium binding domain 1	1.807	0.3916
22974_at	*TPX2*	TPX2, microtubule-associated, homolog (Xenopus laevis)	1.805	0.6156
115330_at	*GPR146*	G protein-coupled receptor 146	1.801	4.9964
79895_at	*ATP8B4*	ATPase, class I, type 8B, member 4	1.794	0.3916

7130_at	*TNFAIP6*	tumor necrosis factor, alpha-induced protein 6	1.794	2.5934
200132_at	*TCTEX1D1*	Tctex1 domain containing 1	1.793	1.7031
286_at	*ANK1*	ankyrin 1, erythrocytic	1.792	4.9964
4635_at	*MYL4*	myosin, light chain 4, alkali; atrial, embryonic	1.788	6.4318
3145_at	*HMBS*	hydroxymethylbilane synthase	1.784	1.7031
7102_at	*TSPAN7*	tetraspanin 7	1.772	6.9774
29128_at	*UHRF1*	ubiquitin-like with PHD and ring finger domains 1	1.771	0.3916
1870_at	*E2F2*	E2F transcription factor 2	1.756	2.2070
701_at	*BUB1B*	budding uninhibited by benzimidazoles 1 homolog beta (yeast)	1.749	0.3916
6036_at	*RNASE2*	ribonuclease, RNase A family, 2 (liver, eosinophil-derived neurotoxin)	1.745	0.3916
2862_at	*MLNR*	motilin receptor	1.739	1.1053
8358_at	*HIST1H3B*	histone cluster 1, H3b	1.734	1.0142
6536_at	*SLC6A9*	solute carrier family 6 (neurotransmitter transporter, glycine), member 9	1.733	4.0068
8825_at	*LIN7A*	lin-7 homolog A (C. elegans)	1.733	0.3916
2200_at	*FBN1*	fibrillin 1	1.729	0.3916
94031_at	*HTRA3*	HtrA serine peptidase 3	1.729	0.3916
3554_at	*IL1R1*	interleukin 1 receptor, type I	1.728	0.3916
6241_at	*RRM2*	ribonucleotide reductase M2	1.727	1.0624
5872_at	*RAB13*	RAB13, member RAS oncogene family	1.726	0.3916
7052_at	*TGM2*	transglutaminase 2 (C polypeptide, protein-glutamine-gamma-glutamyltransferase)	1.724	4.4952
11240_at	*PADI2*	peptidyl arginine deiminase, type II	1.718	0.7929
6622_at	*SNCA*	synuclein, alpha (non A4 component of amyloid precursor)	1.718	2.5934
143689_at	*PIWIL4*	piwi-like 4 (Drosophila)	1.713	0.3916
8447_at	*DOC2B*	double C2-like domains, beta	1.713	0.3916

9787_at	*DLGAP5*	discs, large (Drosophila) homolog-associated protein 5	1.712	0.3916
645_at	*BLVRB*	biliverdin reductase B (flavin reductase (NADPH))	1.707	1.0142
1063_at	*CENPF*	centromere protein F, 350/400ka (mitosin)	1.700	0.3916
51514_at	*DTL*	denticleless homolog (Drosophila)	1.699	1.1053
202243_at	*CCDC125*	coiled-coil domain containing 125	1.697	0.7929
29899_at	*GPSM2*	G-protein signaling modulator 2 (AGS3-like, C. elegans)	1.697	0.3916
983_at	*CDK1*	cyclin-dependent kinase 1	1.695	0.3916
129642_at	*MBOAT2*	membrane bound O-acyltransferase domain containing 2	1.695	1.0624
2996_at	*GYPE*	glycophorin E (MNS blood group)	1.692	4.9964
58528_at	*RRAGD*	Ras-related GTP binding D	1.691	0.3916
3832_at	*KIF11*	kinesin family member 11	1.690	0.7929
55711_at	*FAR2*	fatty acyl CoA reductase 2	1.689	0.6156
1084_at	*CEACAM3*	carcinoembryonic antigen-related cell adhesion molecule 3	1.688	1.0624
84078_at	*KBTBD7*	kelch repeat and BTB (POZ) domain containing 7	1.687	0.6156
56992_at	*KIF15*	kinesin family member 15	1.686	0.3916
4602_at	*MYB*	v-myb myeloblastosis viral oncogene homolog (avian)	1.680	1.0624
8778_at	*SIGLEC5*	sialic acid binding Ig-like lectin 5	1.677	0.6156
259266_at	*ASPM*	asp (abnormal spindle) homolog, microcephaly associated (Drosophila)	1.675	0.6156
5746_at	*PTH2R*	parathyroid hormone 2 receptor	1.675	0.3916
2305_at	*FOXM1*	forkhead box M1	1.675	0.3916
1193_at	*CLIC2*	chloride intracellular channel 2	1.668	4.0068
54855_at	*FAM46C*	family with sequence similarity 46, member C	1.667	6.4318
343171_at	*OR2W3*	olfactory receptor, family 2, subfamily W, member 3	1.662	9.0379
4784_at	*NFIX*	nuclear factor I/X (CCAAT-binding transcription factor)	1.660	1.0624

284415_at	*VSTM1*	V-set and transmembrane domain containing 1	1.658	2.9821
90381_at	*C15orf42*	chromosome 15 open reading frame 42	1.655	0.3916
5055_at	*SERPINB2*	serpin peptidase inhibitor, clade B (ovalbumin), member 2	1.654	0.3916
692087_at	*SNORD49B*	small nucleolar RNA, C/D box 49B	1.654	4.0068
5337_at	*PLD1*	phospholipase D1, phosphatidylcholine-specific	1.653	0.3916
58484_at	*NLRC4*	NLR family, CARD domain containing 4	1.653	0.7929
890_at	*CCNA2*	cyclin A2	1.652	0.6156
79733_at	*E2F8*	E2F transcription factor 8	1.648	0.3916
54458_at	*PRR13*	proline rich 13	1.646	0.3916
3792_at	*KEL*	Kell blood group, metallo-endopeptidase	1.645	9.0379
199675_at	*C19orf59*	chromosome 19 open reading frame 59	1.639	0.3916
55859_at	*BEX1*	brain expressed, X-linked 1	1.638	1.0624
5347_at	*PLK1*	polo-like kinase 1 (Drosophila)	1.631	0.7929
51203_at	*NUSAP1*	nucleolar and spindle associated protein 1	1.626	0.6156
26577_at	*PCOLCE2*	procollagen C-endopeptidase enhancer 2	1.626	0.7929
3009_at	*HIST1H1B*	histone cluster 1, H1b	1.619	0.7929
8395_at	*PIP5K1B*	phosphatidylinositol-4-phosphate 5-kinase, type I, beta	1.617	1.0624
6710_at	*SPTB*	spectrin, beta, erythrocytic	1.617	7.6147
51218_at	*GLRX5*	glutaredoxin 5	1.616	2.9821
9928_at	*KIF14*	kinesin family member 14	1.613	0.3916
3382_at	*ICA1*	islet cell autoantigen 1, 69kDa	1.610	0.3916
55432_at	*YOD1*	YOD1 OTU deubiquinating enzyme 1 homolog (S. cerevisiae)	1.610	2.5934
57556_at	*SEMA6A*	sema domain, transmembrane domain (TM), and cytoplasmic domain, (semaphorin) 6A	1.609	0.6156
26996_at	*GPR160*	G protein-coupled receptor 160	1.609	1.0624

25801_at	*GCA*	grancalcin, EF-hand calcium binding protein	1.603	0.3916
54757_at	*FAM20A*	family with sequence similarity 20, member A	1.603	9.9658
116159_at	*CYYR1*	cysteine/tyrosine-rich 1	1.601	1.0624
272_at	*AMPD3*	adenosine monophosphate deaminase 3	1.596	0.3916
100289137_at	*LOC100289137*	similar to hCG2010820	1.592	3.3639
64101_at	*LRRC4*	leucine rich repeat containing 4	1.583	1.5110
8355_at	*HIST1H3G*	histone cluster 1, H3g	1.579	1.9145
2204_at	*FCAR*	Fc fragment of IgA, receptor for	1.579	0.7929
116151_at	*C20orf108*	chromosome 20 open reading frame 108	1.577	4.4952
11113_at	*CIT*	citron (rho-interacting, serine/threonine kinase 21)	1.571	0.7929
1089_at	*CEACAM4*	carcinoembryonic antigen-related cell adhesion molecule 4	1.570	1.0142
25893_at	*TRIM58*	tripartite motif-containing 58	1.570	4.4952
3772_at	*KCNJ15*	potassium inwardly-rectifying channel, subfamily J, member 15	1.563	5.4721
150468_at	*CKAP2L*	cytoskeleton associated protein 2-like	1.562	1.0624
9493_at	*KIF23*	kinesin family member 23	1.561	0.3916
51734_at	*SEPX1*	selenoprotein X, 1	1.561	0.3916
10158_at	*PDZK1IP1*	PDZK1 interacting protein 1	1.558	3.4989
2352_at	*FOLR3*	folate receptor 3 (gamma)	1.555	9.0379
2995_at	*GYPC*	glycophorin C (Gerbich blood group)	1.553	4.4952
9134_at	*CCNE2*	cyclin E2	1.551	1.0624
100049587_at	*SIGLEC14*	sialic acid binding Ig-like lectin 14	1.551	1.1053
199221_at	*DZIP1L*	DAZ interacting protein 1-like	1.550	0.3916
7855_at	*FZD5*	frizzled homolog 5 (Drosophila)	1.549	0.3916
253827_at	*MSRB3*	methionine sulfoxide reductase B3	1.546	1.1053

9133_at	*CCNB2*	cyclin B2	1.544	0.3916
9055_at	*PRC1*	protein regulator of cytokinesis 1	1.543	1.0142
2934_at	*GSN*	gelsolin (amyloidosis, Finnish type)	1.540	0.3916
150000_at	*ABCC13*	ATP-binding cassette, sub-family C (CFTR/MRP), member 13	1.539	4.9964
6196_at	*RPS6KA2*	ribosomal protein S6 kinase, 90kDa, polypeptide 2	1.539	0.3916
221184_at	*CPNE2*	copine II	1.538	0.3916
167555_at	*FAM151B*	family with sequence similarity 151, member B	1.538	0.3916
6035_at	*RNASE1*	ribonuclease, RNase A family, 1 (pancreatic)	1.534	1.9145
338811_at	*FAM19A2*	family with sequence similarity 19 (chemokine (C-C motif)-like), member A2	1.533	1.1053
285696_at	*LOC285696*	hypothetical LOC285696	1.531	0.3916
25819_at	*CCRN4L*	CCR4 carbon catabolite repression 4-like (S. cerevisiae)	1.530	1.0624
388507_at	*ZNF788*	zinc finger family member 788	1.528	0.7929
10272_at	*FSTL3*	follistatin-like 3 (secreted glycoprotein)	1.527	0.3916
8613_at	*PPAP2B*	phosphatidic acid phosphatase type 2B	1.527	0.3916
56670_at	*SUCNR1*	succinate receptor 1	1.525	2.5934
157570_at	*ESCO2*	establishment of cohesion 1 homolog 2 (S. cerevisiae)	1.524	1.1053
140803_at	*TRPM6*	transient receptor potential cation channel, subfamily M, member 6	1.523	1.9145
699_at	*BUB1*	budding uninhibited by benzimidazoles 1 homolog (yeast)	1.522	1.1053
85462_at	*FHDC1*	FH2 domain containing 1	1.522	8.3194
9086_at	*EIF1AY*	eukaryotic translation initiation factor 1A, Y-linked	1.519	4.9964
3142_at	*HLX*	H2.0-like homeobox	1.519	0.3916
57082_at	*CASC5*	cancer susceptibility candidate 5	1.519	0.3916
2766_at	*GMPR*	guanosine monophosphate reductase	1.518	7.6147
598_at	*BCL2L1*	BCL2-like 1	1.518	5.4721

22824_at	*HSPA4L*	heat shock 70kDa protein 4-like	1.515	1.7031
966_at	*CD59*	CD59 molecule, complement regulatory protein	1.513	1.0624
8794_at	*TNFRSF10C*	tumor necrosis factor receptor superfamily, member 10c, decoy without an intracellular domain	1.511	4.4952
3084_at	*NRG1*	neuregulin 1	1.509	6.4318
2039_at	*EPB49*	erythrocyte membrane protein band 4.9 (dematin)	1.508	9.9658
1647_at	*GADD45A*	growth arrest and DNA-damage-inducible, alpha	1.508	0.3916
84628_at	*NTNG2*	netrin G2	1.504	1.9145
1053_at	*CEBPE*	CCAAT/enhancer binding protein (C/EBP), epsilon	1.502	0.7929
10170_at	*DHRS9*	dehydrogenase/reductase (SDR family) member 9	1.501	1.7031
81563_at	*C1orf21*	chromosome 1 open reading frame 21	-1.501	2.9821
3820_at	*KLRB1*	killer cell lectin-like receptor subfamily B, member 1	-1.501	3.4989
10578_at	*GNLY*	granulysin	-1.502	4.4952
259292_at	*TAS2R46*	taste receptor, type 2, member 46	-1.502	2.9821
917_at	*CD3G*	CD3g molecule, gamma (CD3-TCR complex)	-1.504	2.9821
5243_at	*ABCB1*	ATP-binding cassette, sub-family B (MDR/TAP), member 1	-1.507	2.5934
9832_at	*JAKMIP2*	janus kinase and microtubule interacting protein 2	-1.509	2.9821
256957_at	*C17orf66*	chromosome 17 open reading frame 66	-1.510	2.9821
83988_at	*NCALD*	neurocalcin delta	-1.510	2.9821
81794_at	*ADAMTS10*	ADAM metallopeptidase with thrombospondin type 1 motif, 10	-1.520	2.5934
54843_at	*SYTL2*	synaptotagmin-like 2	-1.520	4.0068
83888_at	*FGFBP2*	fibroblast growth factor binding protein 2	-1.521	3.4989
116987_at	*AGAP1*	ArfGAP with GTPase domain, ankyrin repeat and PH domain 1	-1.528	2.9821
59352_at	*LGR6*	leucine-rich repeat-containing G protein-coupled receptor 6	-1.531	2.9821
4646_at	*MYO6*	myosin VI	-1.534	2.9821

3127_at	*HLA-DRB5*	major histocompatibility complex, class II, DR beta 5	-1.540	3.4989
8115_at	*TCL1A*	T-cell leukemia/lymphoma 1A	-1.542	8.3194
22914_at	*KLRK1*	killer cell lectin-like receptor subfamily K, member 1	-1.544	2.5934
5727_at	*PTCH1*	patched homolog 1 (Drosophila)	-1.554	2.5934
5729_at	*PTGDR*	prostaglandin D2 receptor (DP)	-1.557	3.4989
4603_at	*MYBL1*	v-myb myeloblastosis viral oncogene homolog (avian)-like 1	-1.561	2.9821
343413_at	*FCRL6*	Fc receptor-like 6	-1.567	3.3639
154075_at	*SAMD3*	sterile alpha motif domain containing 3	-1.576	2.9821
55450_at	*CAMK2N1*	calcium/calmodulin-dependent protein kinase II inhibitor 1	-1.577	2.5934
59084_at	*ENPP5*	ectonucleotide pyrophosphatase/phosphodiesterase 5 (putative function)	-1.582	2.9821
3824_at	*KLRD1*	killer cell lectin-like receptor subfamily D, member 1	-1.593	2.5934
401124_at	*DTHD1*	death domain containing 1	-1.594	4.9964
2999_at	*GZMH*	granzyme H (cathepsin G-like 2, protein h-CCPX)	-1.602	3.3639
9289_at	*GPR56*	G protein-coupled receptor 56	-1.605	2.9821
9254_at	*CACNA2D2*	calcium channel, voltage-dependent, alpha 2/delta subunit 2	-1.612	3.3639
7049_at	*TGFBR3*	transforming growth factor, beta receptor III	-1.618	2.9821
8302_at	*KLRC4*	killer cell lectin-like receptor subfamily C, member 4	-1.628	2.9821
22807_at	*IKZF2*	IKAROS family zinc finger 2 (Helios)	-1.672	2.5934
51348_at	*KLRF1*	killer cell lectin-like receptor subfamily F, member 1	-1.674	2.5934
3823_at	*KLRC3*	killer cell lectin-like receptor subfamily C, member 3	-1.688	2.9821
5816_at	*PVALB*	parvalbumin	-1.725	5.4721
11126_at	*CD160*	CD160 molecule	-1.813	2.5934
400360_at	*C15orf54*	chromosome 15 open reading frame 54	-1.871	2.9821
28559_at	*TRBV28*	T cell receptor beta variable 28	-2.021	3.3639

3123_at	*HLA-DRB1*	major histocompatibility complex, class II, DR beta 1	-2.205	3.3639

Supplementary Table S2: Up- and downregulated pathways associated with low overall survival, sorted by number of genes involved.

P-value	N genes involved	N genes at pathway	REACT_ID	Name of the event
			**UP-REGULATED**	
			**Metabolism**	
0.015392694	19	200	REACT_13	Metabolism of amino acids and derivatives
6.30E-005	18	116	REACT_474	Metabolism of carbohydrates
0.000284586	13	77	REACT_1698	Metabolism of nucleotides
7.37E-006	11	40	REACT_1046	Pyruvate metabolism and Citric Acid (TCA) cycle
0.000353746	10	50	REACT_13565	Regulation of ornithine decarboxylase (ODC)
0.001542951	9	50	REACT_1221	CDK-mediated phosphorylation and removal of Cdc6
0.003932045	9	57	REACT_723	Glucose metabolism
0.000302809	6	18	REACT_21330	Synthesis and interconversion of nucleotide di- and triphosphates
0.000422025	6	19	REACT_1785	Citric acid cycle (TCA cycle)
0.007633839	6	32	REACT_522	Purine metabolism
0.000693541	5	14	REACT_1008	Glycogen breakdown (glycogenolysis)
0.002467109	5	18	REACT_2071	Pyruvate metabolism
0.007701138	5	23	REACT_2002	Endosome acidification
0.007701138	5	23	REACT_25268	Acidification of Tf:TfR1 containing endosome
0.000292334	4	7	REACT_1859	Pentose phosphate pathway (hexose monophosphate shunt)
0.00055902	4	8	REACT_1736	Glycogen synthesis
0.006118549	4	14	REACT_9431	Metabolism of porphyrins
0.022740197	4	20	REACT_16957	Peroxisomal lipid metabolism
0.000662594	3	4	REACT_1827	alpha-D-Glucose + ATP => alpha-D-glucose 6-phosphate + ADP

0.000662594	3	4	REACT_1407	Plasmalogen biosynthesis
0.011277931	3	9	REACT_9465	Heme biosynthesis
0.015454102	3	10	REACT_2086	Purine catabolism
0.015454102	3	10	REACT_22099	Phosphorylation of L1 by ERK
0.026077985	3	12	REACT_1923	Purine salvage
0.047672127	3	15	REACT_6759	Formation of ATP by chemiosmotic coupling
0.047672127	3	15	REACT_190	ATP is synthesized from ADP and Pi by ATPase
0.047672127	3	15	REACT_1985	Enzyme-bound ATP is released
0.047672127	3	15	REACT_991	ADP and Pi bind to ATPase
0.003113249	2	2	REACT_2164	alpha-D-Glucose 6-phosphate <=> D-Glucose 1-phosphate
0.003113249	2	2	REACT_605	D-Glucose 1-phosphate <=> alpha-D-Glucose 6-phosphate
0.003113249	2	2	REACT_939	Insulin effects increased synthesis of Xylulose-5-Phosphate
0.003113249	2	2	REACT_9463	Succinyl CoA and glycine condense to form 5-aminolevulinate (ALA)
0.008994111	2	3	REACT_1865	alpha-ketoadipate + CoASH + NAD+ => glutaryl-CoA + CO2 + NADH + H+
0.008994111	2	3	REACT_66	alpha-ketoglutarate + CoASH + NAD+ => succinyl-CoA + CO2 + NADH + H+
0.008994111	2	3	REACT_375	NDP + reduced glutaredoxin => dNDP + oxidized glutaredoxin + H2O
0.008994111	2	3	REACT_2182	NDP + reduced thioredoxin => dNDP + oxidized thioredoxin + H2O
0.008994111	2	3	REACT_642	NDP + reduced thioredoxin => dNDP + oxidized thioredoxin + H2O
0.017325627	2	4	REACT_1053	8 UDP-glucose + ((1,4)-alpha-D-glucosyl)4 glycogenin => 8 UDP + ((1,4)-alpha-D-glucosyl)8 glycogenin [GYS D form]
0.017325627	2	4	REACT_1169	8 UDP-glucose + ((1,4)-alpha-D-glucosyl}4 glycogenin => 8 UDP + ((1,4)-alpha-D-glucosyl)8 glycogenin [GYS I form]
0.017325627	2	4	REACT_16882	Basigin binds MCT1, MCT3 and MCT4
0.017325627	2	4	REACT_14855	lactate + H+ [extracellular] <=> lactate + H+ [cytosol]
0.017325627	2	4	REACT_14839	lactate + H+ [cytosol] <=> lactate + H+ [extracellular]

0.027817417	2	5	REACT_983	pyruvate + CoASH + NAD+ => acetylCoA + CO2 + NADH + H+
0.027817417	2	5	REACT_1623	glycogen phosphorylase (PYGL) dimer b + 2 ATP => glycogen phosphorylase (PYGL) dimer a + 2 ADP
0.027817417	2	5	REACT_13578	Antizyme inhibitor binds to OAZ and stablizes ODC complex
			**Apoptosis**	
0.001471786	17	137	REACT_578	Apoptosis
0.00443228	9	58	REACT_13648	Regulation of Apoptosis
0.007839346	3	8	REACT_1213	Apoptosis induced DNA fragmentation
			**DNA damage/repair**	
0.002053978	9	52	REACT_2160	p53-Independent DNA Damage Response
0.002053978	9	52	REACT_1208	p53-Independent G1/S DNA damage checkpoint
0.003064646	9	55	REACT_1625	p53-Dependent G1 DNA Damage Response
0.003064646	9	55	REACT_85	p53-Dependent G1/S DNA damage checkpoint
0.00443228	9	58	REACT_2254	G1/S DNA Damage Checkpoints
0.007839346	3	8	REACT_13462	Activation of DNA fragmentation factor
0.047672127	3	15	REACT_378	Repair synthesis of patch ~27-30 bases long by DNA polymerase
0.047672127	3	15	REACT_1993	Repair synthesis for gap-filling by DNA polymerase in TC-NER
			**Protein degradation**	
0.002435364	11	74	REACT_9453	Vif-mediated degradation of APOBEC3G
0.002435364	11	74	REACT_9466	Proteosome-mediated degradation of APOBEC3G
0.001581725	10	60	REACT_6715	Degradation of multiubiquitinated Cdh1
0.001581725	10	60	REACT_6785	Autodegradation of Cdh1 by Cdh1:APC/C
0.002608064	10	64	REACT_6871	APC/C:Cdc20 mediated degradation of Securin
0.002608064	10	64	REACT_6777	Degradation of multiubiquitinated Securin
0.002608064	10	64	REACT_9035	APC/C:Cdh1-mediated degradation of Skp2

0.0036828	10	67	REACT_6850	Cdc20:Phospho-APC/C mediated degradation of Cyclin A
0.0036828	10	67	REACT_6937	Degradation multiubiquitinated Cyclin A
0.004110225	10	68	REACT_6826	Degradation of multiubiquitinated cell cycle proteins
0.004575942	10	69	REACT_6761	APC/C:Cdh1 mediated degradation of Cdc20 and other APC/C:Cdh1 targeted proteins in late mitosis/early G1
0.004575942	10	69	REACT_6781	APC/C:Cdc20 mediated degradation of mitotic proteins
0.005082255	10	70	REACT_6954	Activation of APC/C and APC/C:Cdc20 mediated degradation of mitotic proteins
0.011867326	10	79	REACT_6828	APC/C-mediated degradation of cell cycle proteins
0.001139438	9	48	REACT_13491	26S proteosome degrades ODC holoenzyme complex
0.001139438	9	48	REACT_75871	Proteasomal cleavage of substrate
0.001328894	9	49	REACT_1471	Ubiquitinated geminin is degraded by the proteasome
0.001328894	9	49	REACT_480	Ubiquitinated Orc1 is degraded by the proteasome
0.001328894	9	49	REACT_1210	Ubiquitinated Cdc6 is degraded by the proteasome
0.001328894	9	49	REACT_873	Proteolytic degradation of ubiquitinated-Cdc25A
0.001328894	9	49	REACT_2142	Proteasome mediated degradation of Cyclin D1
0.001328894	9	49	REACT_13413	Proteasome mediated degradation of PAK-2p34
0.001328894	9	49	REACT_13464	Regulation of activated PAK-2p34 by proteasome mediated degradation
0.001328894	9	49	REACT_13505	Proteasome mediated degradation of PAK-2p34
0.001328894	9	49	REACT_20637	Proteasome mediated degradation of COP1
0.001542951	9	50	REACT_4	Ubiquitin-dependent degradation of Cyclin D1
0.001542951	9	50	REACT_938	Ubiquitin-dependent degradation of Cyclin D
0.00178386	9	51	REACT_20549	Autodegradation of the E3 ubiquitin ligase COP1
0.002053978	9	52	REACT_1614	Ubiquitin Mediated Degradation of Phosphorylated Cdc25A
0.002053978	9	52	REACT_9034	Degradation of ubiquitinated p27/p21 by the 26S proteasome

0.002053978	9	52	REACT_9003	SCF(Skp2)-mediated degradation of p27/p21
0.002355762	9	53	REACT_75842	Antigen processing: Ubiquitination & Proteasome degradation
0.002691768	9	54	REACT_6821	SCF-beta-TrCP mediated degradation of Emi1
0.002691768	9	54	REACT_6878	SCF-mediated degradation of Emi1
0.003477132	9	56	REACT_11063	Degradation of beta-catenin by the destruction complex
0.003477132	9	56	REACT_10017	Degradation of ubiquitinated -beta catenin by the proteasome
0.024742631	9	76	REACT_9031	Vpu mediated degradation of CD4
0.024742631	9	76	REACT_9018	Degradation of ubiquitinated CD4
0.015454102	3	10	REACT_25315	Ubiquitination of AUF1 (hnRNP D0)
		**Signal transduction**		
0.048274995	18	212	REACT_11061	Signalling by NGF
0.047835111	12	126	REACT_12056	NGF signalling via TRKA from the plasma membrane
0.003477132	9	56	REACT_11045	Signaling by Wnt
0.034044476	6	44	REACT_75913	Nucleotide-binding domain, leucine rich repeat containing receptor (NLR) signaling pathways
0.015395034	5	27	REACT_12484	EGFR downregulation
0.026889844	4	21	REACT_21399	activated TAK1 mediates p38 MAPK activation
0.003044842	3	6	REACT_21395	activated human MKK3/MKK6 phosphorylates p38 MAPK complexed with MAPKAPK2 or MAPKAPK3
0.020385123	3	11	REACT_12435	Retrograde neurotrophin signalling
0.032530817	3	13	REACT_12065	p38MAPK events
0.047672127	3	15	REACT_22240	Phosphorylation of L1 by p90rsk
0.017325627	2	4	REACT_12032	MAP kinase activates MAPKAPK2, MAPKAPK3 and MSK1
0.017325627	2	4	REACT_21375	Active p38 MAPK phosphorylates MAPKAPK2 or 3
0.017325627	2	4	REACT_21358	Nuclear export of human p38 MAPK mediated by its substrate MAPKAPK2 or 3
		**Cell Transport**		

0.000681435	13	84	REACT_11123	Membrane Trafficking
0.001985072	13	94	REACT_6305	Respiratory electron transport, ATP synthesis by chemiosmotic coupling, and heat production by uncoupling proteins.
0.009107485	10	76	REACT_22393	Respiratory electron transport
0.000100291	8	28	REACT_27258	Endosomal Sorting Complex Required For Transport (ESCRT)
0.000532609	7	27	REACT_25283	Transferrin endocytosis and recycling
0.003819463	7	37	REACT_25060	Iron uptake and transport
0.000993223	5	15	REACT_27290	MVB Vesicle Formation
0.001379383	5	16	REACT_27184	ESCRT Disassembly
0.011097932	5	25	REACT_1109	Insulin receptor recycling
0.019006494	4	19	REACT_12495	Assembly in clathrin-coated vesicles (CCVs)
0.007839346	3	8	REACT_22118	L1 binds to AP-2 Clathrin complex
0.011277931	3	9	REACT_12559	Formation of clathrin-coated vesicle
0.011277931	3	9	REACT_22103	Transport of L1 into endosomes
0.011277931	3	9	REACT_22359	Formation of clathrin coated vesicle
0.015454102	3	10	REACT_12397	Endocytosis (internalization) of clathrin-coated vesicle
0.020385123	3	11	REACT_12385	Axonal transport of NGF:Trk complexes
0.020385123	3	11	REACT_22136	Transport of L1 from C-domain to P-domain
			**Cell growth**	
0.00063613	14	94	REACT_2014	Synthesis of DNA
0.001923966	14	105	REACT_899	S Phase
0.003469373	13	100	REACT_1783	G1/S Transition
0.007275887	13	109	REACT_21267	Mitotic G1-G1/S phases
0.011273817	13	115	REACT_1538	Cell Cycle Checkpoints

0.001290635	12	79	REACT_1725	M/G1 Transition
0.001290635	12	79	REACT_734	DNA Replication Pre-Initiation
0.000712619	11	64	REACT_2243	Assembly of the pre-replicative complex
0.001203735	11	68	REACT_1156	Orc1 removal from chromatin
0.001203735	11	68	REACT_2148	Switching of origins to a post-replicative state
0.001538063	11	70	REACT_207	Removal of licensing factors from origins
0.001538063	11	70	REACT_829	Regulation of DNA replication
0.008306703	10	75	REACT_6837	Regulation of APC/C activators between G1/S and early anaphase
0.011867326	10	79	REACT_21279	Regulation of mitotic cell cycle
0.00443228	9	58	REACT_1949	CDT1 association with the CDC6:ORC:origin complex
0.004980802	9	59	REACT_9029	Cyclin A:Cdk2-associated events at S phase entry
0.006234874	9	61	REACT_1574	Cyclin E associated events during G1/S transition
0.034499345	5	33	REACT_7970	Telomere Maintenance
0.003113249	2	2	REACT_936	Formation of Elongin BC complex
0.040203704	2	6	REACT_1303	Mcm4,6,7 trimer forms and associates with the replication fork
0.040203704	2	6	REACT_6922	MCM2-7 mediated fork unwinding
			**Proteins organization**	
0.000857077	13	86	REACT_24994	Regulation of mRNA Stability by Proteins that Bind AU-rich Elements
0.000151167	11	54	REACT_25325	Destabilization of mRNA by AUF1 (hnRNP D0)
0.002053978	9	52	REACT_309	Stabilization of p53
0.00303599	8	45	REACT_25343	Destruction of AUF1 and mRNA
0.017909354	5	28	REACT_75925	Amyloids
0.004568547	4	13	REACT_27319	Cargo Sequestration

0.000172819	3	3	REACT_12073	Heterdimerization of CEACAMs
0.020385123	3	11	REACT_22298	Reinsertion of L1 into the plasma membrane
0.032530817	3	13	REACT_27272	Cargo Recognition And Sorting
0.008994111	2	3	REACT_51	Formation of elongin complex
0.008994111	2	3	REACT_24015	Recruitment of PYK2 to pSIRP alpha
0.008994111	2	3	REACT_21266	Surface deployment of platelet dense granule membrane components
0.027817417	2	5	REACT_75858	Serum amyloid P binds DNA and chromatin
0.040203704	2	6	REACT_25267	Association of AUF1 with Translation and Heat Shock Proteins
		**Inflamation**		
0.003197496	5	19	REACT_75900	Inflammasomes
0.032530817	3	13	REACT_75808	The NLRP3 inflammasome
		**DOWN-REGULATED**		
		**Metabolism**		
0.01065623	14	134	REACT_15550	Insulin Synthesis and Processing
0.005841435	14	125	REACT_11051	Rho GTPase cycle
0.004950053	13	110	REACT_2085	GTP hydrolysis and joining of the 60S ribosomal subunit
0.00229006	12	89	REACT_1654	GTP Hydrolysis by eRF3 bound to the eRF1:mRNA:polypeptide:80S Ribosome complex
0.002076775	12	88	REACT_3	eIF5B:GTP is hydrolyzed and released
0.002076775	12	88	REACT_552	Hydrolysis of eEF1A:GTP
0.002076775	12	88	REACT_13617	MAFA-, NKX2-2-, PAX6-, and PDX1-dependent synthesis of insulin precursor protein
0.00667226	7	43	REACT_15426	PLC beta mediated events
0.021759309	6	42	REACT_12640	Inactivation of Lck by Csk
0.017379403	6	40	REACT_12467	Activation of Lck
0.045583362	4	26	REACT_9000	Calmodulin induced events

0.045583362	4	26	REACT_9053	CaM pathway
0.040180791	4	25	REACT_24941	Circadian Clock
0.030532081	4	23	REACT_18274	Regulation of Insulin Secretion by Glucagon-like Peptide-1
0.030532081	4	23	REACT_24023	Regulation of Water Balance by Renal Aquaporins
0.049064325	3	16	REACT_18342	Nucleotide-like (purinergic) receptors
0.025036396	2	5	REACT_16916	PLC-gamma hydrolyses PIP2
0.015561521	2	4	REACT_23956	PMCA extrusion of Ca2+
0.015561521	2	4	REACT_75931	Trimming of N-ter extended precursor fragments by cytosolic aminopeptidases
		**Apoptosis**		
0.012304399	5	27	REACT_964	Intrinsic Pathway for Apoptosis
0.015561521	2	4	REACT_12563	Interaction and oligomerization of MALT1 to Bcl10
0.015561521	2	4	REACT_13427	NRAGE activates JNK
0.008061587	2	3	REACT_75844	Bcl-2 and Bcl-XL bind NLRP1
		**Immune system**		
0.015561521	2	4	REACT_6786	Ubiquitin-dependent degradation controls basal levels of R-SMAD
0.000186556	60	729	REACT_6900	Immune System
3.23E-005	44	450	REACT_75774	Adaptive Immunity Signaling
0.02307924	22	266	REACT_11152	Immunoregulatory interactions between a Lymphoid and a non-Lymphoid cell
0.004224502	13	108	REACT_13698	Regulation of beta-cell development
0.025267619	12	121	REACT_6167	Influenza Infection
0.018738514	12	116	REACT_6145	Influenza Life Cycle
0.014500061	12	112	REACT_6152	Influenza Viral RNA Transcription and Replication
0.008885861	12	105	REACT_9491	Viral mRNA Translation
0.008247427	12	104	REACT_9514	Viral Protein Synthesis

0.002076775	12	88	REACT_9524	Synthesis of PB1-F2
0.000624275	12	77	REACT_19344	Costimulation by the CD28 family
0.020191091	8	65	REACT_6835	The role of Nef in HIV-1 replication and disease pathogenesis
0.011636903	8	59	REACT_11080	TCR complex interacts with peptide antigen-presenting MHC Class I
0.005093469	7	41	REACT_12596	Translocation of ZAP-70 to Immunological synapse
0.00037935	7	27	REACT_19183	CD28 co-stimulation
0.042758584	6	49	REACT_23853	Interactions of the immunoglobulin superfamily (IgSF) member proteins
0.017561519	3	11	REACT_19238	CD28 dependent Vav1 pathway
0.002593132	3	6	REACT_27145	Interleukin-2: IL2 receptor alpha:beta binds IL2 receptor gamma subunit
0.025036396	2	5	REACT_11121	C3d-complexed antigen binds to complement receptor
0.015561521	2	4	REACT_11119	NKG2A-CD94 heterdimer interacts with HLA-E
0.015561521	2	4	REACT_14847	Receptors CCR3, 4 and 5 bind CCL5 ligand
0.015561521	2	4	REACT_27278	Interleukin-2 receptor alpha:IL2 binds Interleukin-2 receptor beta
0.008061587	2	3	REACT_11106	MHC Class I interacts with CD160
0.008061587	2	3	REACT_11140	KIR2DL1 interacting with HLA-C group 2 (Cw3)
0.008061587	2	3	REACT_11124	KIR3DL2 interacting with HLA-A3
0.002784626	2	2	REACT_19295	SEMA4D interacts with CD45
			**Signal transduction**	
0.001650096	22	212	REACT_11061	Signalling by NGF
1.22E-006	17	84	REACT_12526	TCR signaling
0.005841435	14	125	REACT_11044	Signaling by Rho GTPases
0.015027192	13	126	REACT_12056	NGF signalling via TRKA from the plasma membrane
0.004950053	13	110	REACT_75891	Phosphorylated UPF1 Recruits SMG5, SMG7, SMG6, and PP2A

0.003030769	13	104	REACT_75910	SMG1 Phosphorylates UPF1 (Enhanced by Exon Junction Complex)
0.002780132	13	103	REACT_15421	Cleavage of the Signal Peptide of Preproinsulin
0.000975634	13	92	REACT_12051	Cell surface interactions at the vascular wall
0.005134165	12	98	REACT_15358	Interaction between SRP and SRP Receptor
0.004336652	12	96	REACT_15319	Signal Recognition (Preproinsulin)
0.004336652	12	96	REACT_20565	Signal Recognition (Preprolactin)
0.000759184	11	68	REACT_12555	Downstream TCR signaling
7.65E-005	11	53	REACT_12623	Generation of second messenger molecules
1.86E-005	11	46	REACT_12498	Phosphorylation of PLC-gamma1
0.039060641	9	87	REACT_13776	p75 NTR receptor-mediated signalling
0.00433298	9	61	REACT_18407	G alpha (12/13) signalling events
0.003867925	9	60	REACT_15295	Opioid Signalling
0.000389117	9	44	REACT_12615	Phosphorylation of SLP-76
0.018523772	8	64	REACT_13720	Cell death signalling via NRAGE, NRIF and NADE
0.010525854	8	58	REACT_10098	GEFs activate Rho GTPase:GDP
0.002850499	8	47	REACT_13638	NRAGE signals death through JNK
0.001345375	8	42	REACT_12421	Phosphorylation of TBSMs in LAT
0.009685997	7	46	REACT_23837	Interleukin-3, 5 and GM-CSF signaling
0.007585877	7	44	REACT_15526	G-protein mediated events
0.00667226	7	43	REACT_12582	Phosphorylation of CD3 and TCR zeta chains
0.005843087	7	42	REACT_19216	GEFs activate RhoA,B,C
0.005093469	7	41	REACT_12538	Phosphorylation of ZAP-70 by Lck
0.005093469	7	41	REACT_12394	Activation of ZAP-70

0.005093469	7	41	REACT_12446	Dephosphorylation of Lck-pY505 by CD45
0.001672101	7	34	REACT_12079	PLC-gamma1 signalling
0.03262897	6	46	REACT_19146	Dephosphorylation of CD3-zeta by PD-1 bound phosphatases
0.03262897	6	46	REACT_19324	PD-1 signaling
0.026830065	6	44	REACT_75913	Nucleotide-binding domain, leucine rich repeat containing receptor (NLR) signaling pathways
0.017379403	6	40	REACT_12633	Phosphorylation of ITAM motifs in CD3 complexes
0.017379403	6	40	REACT_13694	p75NTR indirectly activates RAC and Cdc42 via a guanyl-nucleotide exchange factor
0.001556972	6	25	REACT_2202	Effects of PIP2 hydrolysis
0.000155659	6	17	REACT_19358	CD28 dependent PI3K/Akt signaling
0.043360304	5	37	REACT_20563	Activation of NMDA receptor upon glutamate binding and postsynaptic events
0.039123685	5	36	REACT_17025	Down-stream signal transduction
0.027980085	5	33	REACT_1665	Glucagon signaling in metabolic regulation
0.027980085	5	33	REACT_20593	Post NMDA receptor activation events
0.030532081	4	23	REACT_12034	Signaling by BMP
0.030532081	4	23	REACT_23832	Nephrin interactions
0.030532081	4	23	REACT_24024	Gab2 binds the p85 subunit of Class 1A PI3 kinases
0.006557378	4	15	REACT_6844	Signaling by TGF beta
0.000778848	4	9	REACT_19212	PI3K binds CD28
0.000778848	4	9	REACT_19274	CD28 bound PI3K phosphorylates PIP2 to PIP3
0.000105283	4	6	REACT_19159	Gads binds CD28
0.049064325	3	16	REACT_15530	PKA activation
0.041437086	3	15	REACT_19333	G alpha (z) signalling events
0.034461145	3	14	REACT_697	Activation of BH3-only proteins

0.034461145	3	14	REACT_22351	DCC mediated attractive signaling
0.022516538	3	12	REACT_12519	PECAM1 interactions
0.022516538	3	12	REACT_18289	P2Y receptors
0.013283338	3	10	REACT_12013	I-Smad binds to type I receptor, preventing Smad1/5/8 from being activated
0.006707393	3	8	REACT_19264	Phosphorylation of CD28
0.006707393	3	8	REACT_19243	Activation of Rac1 by pVav1
0.006707393	3	8	REACT_19286	Activation of Cdc42 by pVav1
0.006707393	3	8	REACT_25061	Binding of CRY:PER Heterodimer to the BMAL1:CLOCK/NPAS2 Heterodimer
0.006707393	3	8	REACT_27154	Recruited STAT5 is phosphorylated
0.006707393	3	8	REACT_27183	Phosphorylation of IL2RB Y338, Y392 or Y510 enables STAT recruitment
0.006707393	3	8	REACT_27314	Phosphorylated STAT5 is released
0.004361389	3	7	REACT_19316	Activation of Vav1
0.004361389	3	7	REACT_27317	Phosphorylation of IL2RB Y338 enables SHC recruitment
0.004361389	3	7	REACT_27150	SHC1 bound to IL2 receptor is phosphorylated
0.004361389	3	7	REACT_27233	SYK is a substrate for JAK1
0.004361389	3	7	REACT_27313	Syk binds IL2RB
0.002593132	3	6	REACT_19254	Grb2 binds CD28
0.002593132	3	6	REACT_27274	Within the IL-2R complex JAK3 phosphorylates JAK1
0.002593132	3	6	REACT_27196	JAK1 phosphorylates Y338, Y392 and Y510 of IL2RB
0.049016684	2	7	REACT_637	G12/13 activation by PAR
0.049016684	2	7	REACT_23839	Thrombin-activated PAR binds G-protein G12/13
0.049016684	2	7	REACT_23796	Dissociation of the PAR:G12/13 complex
0.036258028	2	6	REACT_6744	Phospho-R-SMAD dissociates from the receptor complex

0.036258028	2	6	REACT_6879	Activated type I receptor phosphorylates R-SMAD directly
0.036258028	2	6	REACT_12070	Type II receptor phosphorylates type I receptor
0.036258028	2	6	REACT_12011	BMP2 binds to the receptor complex
0.036258028	2	6	REACT_12408	Phoshorylation of PECAM-1 by Fyn or Lyn or c-Src
0.036258028	2	6	REACT_16997	Nck binds to the active PDGF receptor
0.036258028	2	6	REACT_20653	ADP signalling through P2Y purinoceptor 12
0.036258028	2	6	REACT_20523	Activated P2Y purinoceptor 12 binds G-protein Gi
0.036258028	2	6	REACT_20507	Gi activation by P2Y purinoceptor 12
0.036258028	2	6	REACT_23820	Nephrin mediated activation of N-WASP
0.025036396	2	5	REACT_16998	PLC-gamma binds to the active receptor
0.025036396	2	5	REACT_12561	PI3K phosphorylates PIP2 to PIP3
0.025036396	2	5	REACT_23958	Activation of beta-arrestin-1-bound Src kinase
0.025036396	2	5	REACT_23900	Activated Src activates ERK
0.025036396	2	5	REACT_23781	Beta-arrestin-1 acts as scaffold for a PAR1 signalling complex
0.025036396	2	5	REACT_18416	S1P-binding receptors bind S1P
0.015561521	2	4	REACT_6887	SKI complexes with the SMAD complex, suppressing TGF-beta signaling
0.015561521	2	4	REACT_23917	Activation of Protein Kinase C novel isoforms
0.008061587	2	3	REACT_1144	Binding of IP3 to IP3 receptor
0.008061587	2	3	REACT_12074	Release of calcium from intracellular stores by IP3 receptor activation
0.008061587	2	3	REACT_12573	Phosphorylation of CARMA1
0.008061587	2	3	REACT_18395	Opening of ER calcium channels by activated PKA
0.008061587	2	3	REACT_18335	Phosphorylation of FANCI by ATM/ATR
0.008061587	2	3	REACT_18293	Phosphorylation of FANCD2 by ATR/ATM

0.008061587	2	3	REACT_25029	Phosphorylation and Nuclear Translocation of the BMAL1:CLOCK/NPAS2 Heterodimer
0.008061587	2	3	REACT_23803	Syk/Lck phosphorylate LAT
0.008061587	2	3	REACT_22135	Inactive G alpha (z) reassociates with G beta:gamma
0.002784626	2	2	REACT_23835	Activated PAR1 binds Beta-arrestin-1
0.002784626	2	2	REACT_27175	IL2RB associates with JAK1
0.008061587	2	3	REACT_12008	IP3 binds to the IP3 receptor, opening the endoplasmic reticulum Ca2+ channel
		**Gene Expression**		
0.043587007	30	413	REACT_71	Gene Expression
0.035911088	20	247	REACT_21257	Metabolism of RNA
0.01276809	20	222	REACT_20605	Metabolism of mRNA
0.005438258	14	124	REACT_1014	Translation
0.003201673	14	117	REACT_2159	Eukaryotic Translation Initiation
0.003201673	14	117	REACT_2099	Cap-dependent Translation Initiation
0.004950053	13	110	REACT_75886	Nonsense-Mediated Decay
0.004950053	13	110	REACT_75822	Nonsense Mediated Decay Enhanced by the Exon Junction Complex
0.004575691	13	109	REACT_79	L13a-mediated translational silencing of Ceruloplasmin expression
0.004575691	13	109	REACT_1762	3' -UTR-mediated translational regulation
0.003895432	13	107	REACT_75787	SMG6 Cleaves mRNA with Premature Termination Codon
0.003030769	13	104	REACT_75753	UPF1 Binds an mRNP with a Termination Codon Preceding an Exon Junction Complex
0.001940961	13	99	REACT_13819	Regulation of gene expression in beta cells
0.005134165	12	98	REACT_1797	Formation of a pool of free 40S subunits
0.003329699	12	93	REACT_75917	Formation of UPF1:eRF3 Complex on mRNA with a Premature Termination Codon and No Exon Junction Complex
0.003329699	12	93	REACT_75768	Nonsense Mediated Decay Independent of the Exon Junction Complex

0.003039637	12	92	REACT_1477	Eukaryotic Translation Elongation
0.00229006	12	89	REACT_1986	Eukaryotic Translation Termination
0.00229006	12	89	REACT_389	Polypeptide release from the eRF3-GDP:eRF1:mRNA:80S Ribosome complex
0.00229006	12	89	REACT_227	GTP bound eRF3:eRF1 complex binds the peptidyl tRNA:mRNA:80S Ribosome complex
0.00229006	12	89	REACT_1404	Peptide chain elongation
0.00229006	12	89	REACT_1937	Translocation of ribosome by 3 bases in the 3' direction
0.002076775	12	88	REACT_198	The 60S subunit joins the translation initiation complex
0.002076775	12	88	REACT_2075	Aminoacyl-tRNA binds to the ribosome at the A-site
0.001880047	12	87	REACT_928	Release of 40S and 60S subunits from the 80S ribosome
0.001880047	12	87	REACT_1227	Peptide transfer from P-site tRNA to the A-site tRNA
0.018513126	7	52	REACT_940	Dissociation of L13a from the 60s ribosomal subunit
0.03262897	6	46	REACT_20639	Deadenylation-dependent mRNA decay
0.004980284	5	22	REACT_20514	Deadenylation of mRNA
0.035164041	4	24	REACT_1309	RNA Polymerase I Transcription
0.026282007	4	22	REACT_1974	RNA Polymerase I Promoter Clearance
0.022408966	4	21	REACT_953	RNA Polymerase I Transcription Initiation
0.022408966	4	21	REACT_1074	RNA Polymerase I Transcription Termination
0.022408966	4	21	REACT_513	Dissociation of PTRF:Polymerase I/Nascent Pre rRNA Complex:TTF-I:Sal Box
0.022408966	4	21	REACT_211	PTRF Binds the Polymerase I Transcription Complex/Nascent Pre rRNA Complex paused at the TTF-I:Sal Box
0.018906088	4	20	REACT_139	Recruitment of Active RNA Polymerase I to SL1:phos.UBF-1:rDNA Promoter
0.018906088	4	20	REACT_373	Loss of Rrn3 from RNA Polymerase I promoter escape complex
0.018906088	4	20	REACT_1913	RNA Polymerase I Promoter Escape
0.018906088	4	20	REACT_2228	Polymerase I Transcription Complex/Nascent Pre rRNA Complex pauses at the TTF-I:Sal Box

0.01576443	4	19	REACT_2204	RNA Polymerase I Chain Elongation
0.01576443	4	19	REACT_872	Elongation of pre-rRNA transcript
0.01576443	4	19	REACT_20651	Deadenylation of mRNA by the CCR4-NOT Complex
0.049016684	2	7	REACT_1173	Binding of Rrn3 to RNA Polymerase I
0.036258028	2	6	REACT_1773	Assembly of RNA Polymerase I Holoenzyme (human)
			**Proteins organization**	
0.004224502	13	108	REACT_15520	Translocation of Preproinsulin to Endoplasmic Reticulum
0.005093469	7	41	REACT_12566	Change of PKC theta conformation
0.005093469	7	41	REACT_12642	Recruitment of ZAP-70 to phosphorylated ITAMs
0.022516538	3	12	REACT_22398	Recruitment of ABLIM to the plasma membrane
0.004361389	3	7	REACT_19244	Translocation of Vav1 to CD28
0.001349207	3	5	REACT_25234	Stabilization of Unphosphorylated BMAL1:CLOCK/NPAS2 Heterodimer by CRY Proteins
0.000561654	3	4	REACT_12506	Recruitment of PLC-gamma1 to SLP-76
0.000561654	3	4	REACT_12604	Recruitment of ITK to SLP-76
0.000561654	3	4	REACT_12525	Disassociation of PLC-gamma1 from SLP-76
0.049016684	2	7	REACT_549	Activation of BAD and translocation to mitochondria
0.049016684	2	7	REACT_1532	cAMP induces dissociation of inactive PKA tetramers
0.036258028	2	6	REACT_6923	An anchoring protein, SARA, recruits R-SMAD
0.036258028	2	6	REACT_11173	ICAMs 1-4 bind to Integrin LFA-1
0.036258028	2	6	REACT_20630	Dissociation of the P2Y purinoceptor 12:Gi complex
0.036258028	2	6	REACT_22150	Liganded Gz-activating GPCRs bind inactive heterotrimeric G-protein Gz
0.036258028	2	6	REACT_22380	Liganded Gz-activating GPCR acts as a GEF for Gz
0.036258028	2	6	REACT_22170	The Ligand:GPCR:Gz complex dissociates

0.025036396	2	5	REACT_12486	Recruitment of PI3K to plasmamembrane
0.025036396	2	5	REACT_12448	Translocation of TRAF6 to CBM complex
0.025036396	2	5	REACT_12009	Formation of a heteromeric BMP receptor complex
0.025036396	2	5	REACT_23852	Nephrin binds NCK
0.015561521	2	4	REACT_701	Activation, myristolyation of BID and translocation to mitochondria
0.008061587	2	3	REACT_11161	VCAM-1interacts with VLA-4
0.008061587	2	3	REACT_11199	MADCAM interacts with Integrin alpha4beta7
0.008061587	2	3	REACT_12489	Recruitment of SLP-76 to Gads
0.008061587	2	3	REACT_12040	VLA-4 binds JAM-B
0.008061587	2	3	REACT_13818	Interaction of integrin alpha4beta1 with Fibronectin
0.008061587	2	3	REACT_13516	Interaction of integrin alpha4beta1 with Thrombospondin-1
0.008061587	2	3	REACT_13810	Interaction of integrin alpha4beta1 with Osteopontin
0.008061587	2	3	REACT_25402	Formation of BMAL1:CLOCK/NPAS2 Heterodimer
0.002784626	2	2	REACT_12398	Recruitment of PLC-gamma1 to LAT
0.002784626	2	2	REACT_12570	Recruitment of Gads to LAT
0.002784626	2	2	REACT_12618	Disassociation of PLC-gamma1 from LAT
			**Inflamation**	
0.01576443	4	19	REACT_75900	Inflammasomes
0.008061587	2	3	REACT_75927	The NLRP1 inflammasome
			**Hemostasis**	
0.000777173	38	426	REACT_604	Hemostasis
0.002969268	23	236	REACT_20	Formation of Platelet plug
0.002360124	22	218	REACT_798	Platelet Activation
0.020191091	8	65	REACT_622	Platelet activation triggers

0.010525854	8	58	REACT_23876	Platelet homeostasis
0.01576443	4	19	REACT_23905	Platelet calcium homeostasis
0.008061587	2	3	REACT_945	Transport of Ca++ from platelet dense tubular system to cytoplasm

Supplementary Table S3: Overexpressed common elements between samples with ≥5 CTCs and samples from patients with shortest survival.

Overexpressed common elements in samples with ≥5 CTCs and shortest survival

	Expression in ≥5 CTCs *vs* <5 CTCS		Overall survival			Expression in ≥5 CTCs *vs* <5 CTCS		Overall survival	
	FC	FDR	HR	FDR		FC	FDR	HR	FDR
ABCA13	3.12	1.06	1.64	0.000	*HIST1H3B*	1.73	1.01	3.50	0.000
AHSP	3.11	2.59	1.38	0.000	*HLX*	1.52	0.39	5.69	0.000
ALAS2	2.84	4.01	1.39	0.085	*HP*	4.09	0.39	1.68	0.000
AMPD3	1.60	0.39	5.58	0.000	*LCN2*	2.28	1.01	2.05	0.000
ANXA3	2.82	1.06	1.72	0.000	*LIN7A*	1.73	0.39	4.63	0.000
ATP8B4	1.79	0.39	3.96	0.000	*LOC643332*	1.96	0.39	3.39	0.000
BCL2L1	1.52	5.47	1.88	0.149	*LTF*	2.78	1.91	1.66	0.000
BLVRB	1.71	1.01	2.30	0.000	*MBOAT2*	1.69	1.06	2.95	0.000
BPGM	1.83	4.50	1.65	0.000	*MGAM*	2.24	1.11	1.80	0.000
BPI	2.45	1.70	1.82	0.000	*MMP8*	3.01	1.70	1.76	0.000
C19orf59	1.64	0.39	5.43	0.000	*MMP9*	3.29	0.79	1.59	0.000
C5orf32	1.88	0.39	3.42	0.000	*MPO*	2.32	1.91	1.91	0.000
CA1	2.84	4.50	1.41	0.149	*MS4A3*	2.12	1.70	2.08	0.000
CCDC125	1.70	0.79	3.04	0.000	*NA*	2.32	0.39	2.88	0.000
CD177	3.27	0.62	1.68	0.000	*NLRC4*	1.65	0.79	3.58	0.000
CD24	2.36	1.01	2.02	0.000	*NTNG2*	1.50	1.91	3.45	0.000
CEACAM1	2.69	1.01	1.80	0.000	*OLFM4*	2.85	2.21	1.69	0.000
CEACAM6	2.40	2.59	1.80	0.000	*PADI2*	1.72	0.79	3.79	0.000
CEACAM8	2.51	2.34	1.77	0.000	*PADI4*	1.90	0.62	2.94	0.000
CHI3L1	3.58	0.39	1.71	0.000	*PGLYRP1*	2.51	1.11	1.90	0.000
CPNE2	1.54	0.39	6.66	0.000	*PLD1*	1.65	0.39	7.57	0.000
CRISP3	3.42	1.06	1.59	0.000	*PRTN3*	2.37	0.62	2.04	0.000
CTSG	2.44	1.91	1.87	0.000	*RNASE2*	1.74	0.39	6.41	0.000
CYP4F3	2.89	0.62	1.70	0.000	*RNASE3*	2.16	1.51	2.10	0.000
DCAF12	1.93	1.51	1.77	0.000	*RRAGD*	1.69	0.39	5.33	0.000
DEFA4	2.33	4.01	1.83	0.000	*S100P*	2.30	1.06	2.06	0.000
EIF1AY	1.52	5.00	1.84	0.085	*SELENBP1*	2.81	2.59	1.40	0.000
ELANE	2.55	1.11	1.89	0.000	*SEPX1*	1.56	0.39	5.20	0.000
EPB49	1.51	9.97	1.74	0.542	*SERPINB10*	2.73	0.62	2.05	0.000
FAM151B	1.54	0.39	4.92	0.000	*SIGLEC5*	1.68	0.62	2.68	0.255
FAM46C	1.67	6.43	1.67	0.085	*SLC25A37*	1.99	1.51	1.76	0.000
FAR2	1.69	0.62	3.53	0.000	*SLC25A39*	1.90	2.59	1.69	0.000
FCAR	1.58	0.79	3.63	0.000	*SLC4A1*	3.28	2.59	1.36	0.000
GCA	1.60	0.39	5.76	0.000	*SNCA*	1.72	2.59	1.71	0.367
GMPR	1.52	7.61	1.74	1.544	*TCN1*	2.76	1.11	1.79	0.000
GSN	1.54	0.39	4.49	0.000	*TRIM58*	1.57	4.50	1.93	0.085
GYPC	1.55	4.50	1.94	0.000	*UGCG*	1.89	0.39	3.25	0.000
HBD	2.27	2.34	1.52	0.000	*VSTM1*	1.66	2.98	2.98	0.085
HIST1H1B	1.62	0.79	5.24	0.000	*YOD1*	1.61	2.59	1.98	0.000

Downexpressed common elements in samples with ≥5 CTCs and shortest survival

	Expression in ≥5 CTCs *vs* <5 CTCS		Overall survival			Expression in ≥5 CTCs *vs* <5 CTCS		Overall survival	
	FC	FDR	HR	FDR		FC	FDR	HR	FDR
ADAMTS10	-1.52	2.59	0.23	0.542	*KLRC3*	-1.69	2.98	0.52	1.125

C1orf21	-1.50	2.98	0.33	0.255	*KLRC4*	-1.63	2.98	0.44	0.754
CD160	-1.81	2.59	0.40	0.367	*KLRD1*	-1.59	2.59	0.40	0.542
CD3G	-1.50	2.98	0.37	0.255	*KLRF1*	-1.67	2.59	0.38	0.367
FCRL6	-1.57	3.36	0.44	0.754	*KLRK1*	-1.54	2.59	0.25	0.149
FGFBP2	-1.52	3.50	0.50	1.544	*MYBL1*	-1.56	2.98	0.32	0.149
GNLY	-1.50	4.50	0.48	0.754	*PTCH1*	-1.55	2.59	0.36	0.754
GPR56	-1.60	2.98	0.47	0.367	*PTGDR*	-1.56	3.50	0.50	1.125
GZMH	-1.60	3.36	0.48	0.754	*PVALB*	-1.73	5.47	0.50	0.367
IKZF2	-1.67	2.59	0.23	0.097	*SAMD3*	-1.58	2.98	0.31	0.149
KLRB1	-1.50	3.50	0.27	0.097	*TGFBR3*	-1.62	2.98	0.39	0.255
